# Inhibition of USP2 eliminates cancer stem cells and enhances TNBC responsiveness to chemotherapy

**DOI:** 10.1038/s41419-019-1512-6

**Published:** 2019-03-28

**Authors:** Jiabei He, Hong-Jen Lee, Suchandrima Saha, Diane Ruan, Hua Guo, Chia-Hsin Chan

**Affiliations:** 1 Department of Pharmacological Sciences, Stony Brook University, Stony Brook, NY 11794 USA; 2 Stony Brook Cancer Center, Stony Brook University, Stony Brook, NY 11794 USA; 3 Department of Pathology and Cell Biology, Columbia University Medical Center, New York, NY 10032 USA

## Abstract

Triple-negative breast cancer (TNBC) is the most aggressive subtype of breast cancer that harbors enriched cancer stem cell (CSC) populations in tumors. Conventional chemotherapy is a standard treatment for TNBC, but it spares the CSC populations, which cause tumor recurrence and progression. Therefore, identification of the core molecular pathway that controls CSC activity and expansion is essential for developing effective therapeutics for TNBC. In this study, we identify that USP2 deubiquitinating enzyme is upregulated in CSCs and is a novel regulator of CSCs. Genetic and pharmacological targeting of USP2 substantially inhibits the self-renewal, expansion and chemoresistance of CSCs. We show that USP2 maintains the CSC population by activating self-renewing factor Bmi1 and epithelial-mesenchymal transition through Twist upregulation. Mechanistically, USP2 promotes Twist stabilization by removing β-TrCP-mediated ubiquitination of Twist. Animal studies indicate that pharmacological inhibition of USP2 suppresses tumor progression and sensitizes tumor responses to chemotherapy in TNBC. Furthermore, the histological analyses reveal a positive correlation between USP2 upregulation and lymph node metastasis. Our findings together demonstrate a previously unrecognized role of USP2 in mediating Twist activation and CSC enrichment, suggesting that targeting USP2 is a novel therapeutic strategy to tackle TNBC.

## Introduction

Treatment of triple-negative breast cancer (TNBC) remains challenging due to lack of effective targeted therapies, chemoresistance and high propensity toward metastasis^1^. Advanced genomic profiling of TNBC has shown that TNBC is enriched for cancer stem cells (CSCs)^2^. CSCs possess unlimited self-renewing and multipotency capacity that allows very few CSCs, including those post-treatment remnants, to give rise to differentiated cancer cell progeny and ultimately regrow heterogeneous tumors at the original (tumor recurrence) and/or distant organs (tumor metastases)^3,4^. In TNBC patients, front-line chemotherapy effectively suppresses the majority of primary tumors by eliminating proliferating cells but often fails to target the slow-cycling CSCs. Identifying molecular drivers and signaling pathways that underlie the self-renewal and expansion of CSCs have the potential to offer new treatment options for this lethal disease.

Ubiquitination is a post-translational modification that attaches various kinds of ubiquitin molecules to protein substrates for regulating protein functions^5^. Diverse ubiquitin chains direct substrates toward different biological outcomes. Lysine (K) 48-linked ubiquitination, the most abundant polyubiquitination form in mammalian cells, targets proteins for proteasome-mediated degradation. On the other hand, K63-linked ubiquitination generally serves as a molecular platform that recruits adapter proteins for modulating protein trafficking, signaling transduction, endocytosis and lysosomal degradation. Aside from protein substrates, Liu et al., recently uncovered that the K63-linked ubiquitin chains can directly interact with DNA via its DNA-binding motif to facilitate DNA repair^6^. Protein ubiquitination catalyzed by E3 ligases can be reversed by deubiquitinating enzymes (DUBs). The human genome encodes at least 100 DUBs. Several DUBs are deregulated in human cancers^7^. There is an expanding list of DUBs proven to play essential roles in orchestrating biological processes related to cancer^8^. For example, UCH5L, USP1, USP3, USP7, USP17 and USP22 are shown to regulate the expression and/or activation of oncoproteins and hence are regarded as attractive targets for anticancer therapy^9–11^. Despite the increasing awareness of the involvement of DUBs in cancer development, the roles of DUBs in regulating CSCs especially in TNBC remain largely unexplored.

Twist is a basic helix–loop–helix transcription factor whose expression is repressed in normal tissues but found to be highly expressed in basal-like TNBC as well as in a wide array of metastatic cancers^12,13^. Twist is an indispensable regulator of CSC self-renewal. A vital mechanism by which Twist enhances CSC properties is the acquisition of mesenchymal phenotype through the epithelial-mesenchymal transition (EMT) process^14,15^. Accumulating evidence indicates that Twist can also orchestrate CSC capacities through EMT-independent manner^16^. Bmi1 is a Polycomb complex protein that controls self-renewal and pluripotency of stem cells and CSCs^17,18^. Previous reports have demonstrated that Twist directly activates Bmi1 by inducting Bmi1 gene transcription^19,20^. These studies highlight the versatile roles of Twist in CSC regulation. Twist has long been perceived as a difficult drug target due to the absence of a ligand-binding domain. Therefore, it is of immense interest to decipher the regulatory machinery and mechanisms responsible for Twist protein expression and induction.

Twist is a short-lived protein since it is rapidly degraded by the ubiquitin-proteasome pathway. β-TrCP and FBXL14 E3 ligases have been identified to induce K48-linked ubiquitination and subsequent protein degradation of Twist. We recently discovered and reported that Twist undergoes K63-linked ubiquitination and this modification enhances Twist stability by preventing the occurrence of K48-linked ubiquitination on Twist^21,22^. In this study, we identified that ubiquitin-specific protease 2 (USP2) deubiquitinating enzyme is a novel activator of Twist and CSCs. We showed that USP2 stabilizes Twist expression by diminishing the ubiquitination-mediated proteasomal degradation of Twist. Our study demonstrated that USP2 is required for the activation of Twist/Bmi1 pathway and Twist-mediated CSC properties. Genetic and pharmacological inhibition of USP2 displays a profound synergy with chemotherapeutic agents on CSC elimination. Animal studies show that the USP2 inhibitor significantly suppresses tumor growth and increases chemotherapy efficacy in TNBC. Furthermore, the histological analyses uncover that USP2 protein upregulation is correlated with lymph node metastasis of breast tumor. Our work reveals that USP2-mediated Twist/Bmi1 pathway represents a cell-intrinsic mechanism crucial for CSC regulation besides EMT and provides pre-clinical evidence that targeting USP2 is indeed a promising approach for anti-CSC therapy.

## Results

### USP2 gene is upregulated in TNBC and is essential for the expansion of CSCs in TNBC

**Fig. 1:**
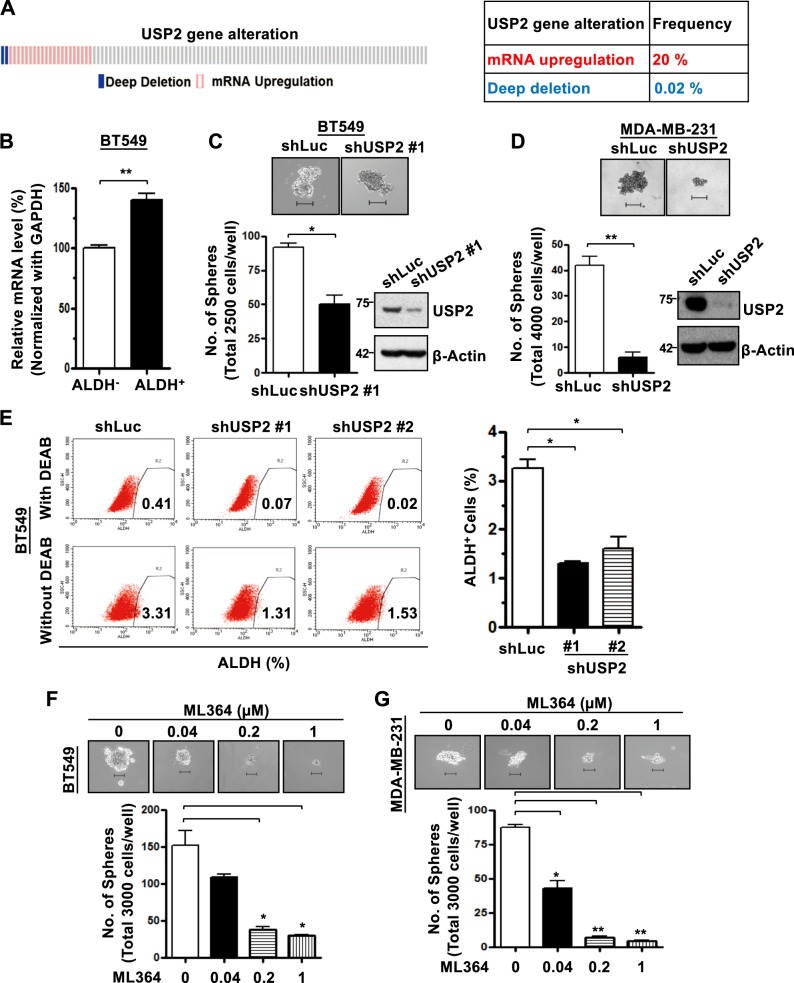
USP2 is upregulated and is essential for CSC self-renewal and expansion in TNBC. **a** cBioportal was used to access TCGA data for USP2 gene alternation from invasive carcinoma of basal-like breast cancer. **b** Real-time PCR analysis of USP2 mRNA levels in the non-CSC (ALDH^−^) and CSC (ALDH^+^) populations isolated from BT549 cells showed that USP2 gene expression is upregulated in CSCs. **c**, **d** Representative images and quantitative results of tumorsphere formation assay in BT549 (**c**), and MDA-MB-231 (**d**) cells stably infected with lentivirus containing shRNA targeting Luciferase or USP2. Western blot results showing the USP2 protein expression in Luciferase and USP2-knockdown cells. These experiments were performed with pools of cell after viral infection, followed by puromycin selection. Scale bar, 100 μm. **e** Representative flow cytometry images of and quantification results for the population of ALDH^+^ cells in Luciferase and USP2-knockdown BT549 cells was determined by flow cytometry analysis. N, N-diethylaminobenzaldehyde (DEAB), an inhibitor of ALDH enzyme, was used as a negative control. **f**, **g** Representative images and quantitative results of tumorsphere formation assay treated with vehicle control (DMSO) or various doses of the USP2 inhibitor ML364 in BT549 (**f**) and MDA-MB-231 (**g**) cells for 7 and 5 days, respectively. Cells were pre-treated with ML364 for 24 h before seeded. Scale bar, 100 μm. Results shown in (**b**–**g**) are presented as mean value ± SEM; *p < 0.05, **p < 0.01

Advanced genomic profiling and hierarchical clustering have shown that breast tumors consist of at least five molecular subtypes: basal-like, human epidermal growth factor receptor 2 (HER2)-enriched, normal-like, luminal A and luminal B^23^. Our bioinformatics analysis of The Cancer Genome Atlas (TCGA) dataset by cBioPortal revealed that the USP2 gene is upregulated in around 20% of basal-like breast tumors (Fig. 1a). Since most (>85%) TNBC samples were classified into the basal-like subtype^24,25^, this observation led us to interrogate the potential pathological role of USP2 in basal-like TNBC. TNBC is the most aggressive subtype of breast cancer featuring CSC enrichment^2^. A desirable approach for developing effective anticancer therapies against TNBC is to target CSCs, which are characterized by high activity of aldehyde dehydrogenase (ALDH). ALDH is a well-established marker for identifying and isolating CSCs from various tissue sources^26^. We isolated the CSC populations based on ALDH activity from BT549, a basal-like TNBC cell line, and found that USP2 expression in the CSC population of TNBC is significantly higher than that in non-CSCs (Fig. 1b). We subsequently investigated the effect of USP2 on CSC activity. Tumorsphere cultivation is widely-used as a functional approach to study the self-renewal activity of CSCs^27^. Tumorsphere formation assays demonstrated that the overexpression of USP2 increases the formation and quantity of tumorspheres in three basal-like TNBC cell models—BT549, MDA-MB-231 and MDA-MB-157 (Supplementary Figure 1a-c). Moreover, the enzymatically inactive mutant of USP2 failed to promote CSC renewal, suggesting that the CSC-promoting effect mediated by USP2 depends on its deubiquitinase activity (Supplementary Figure 1c). Conversely, knocking down USP2 expression reduced the size and number of tumorspheres in various TNBC cell lines (Fig. 1c, d and Supplementary Figure 2a–d). In line with these observations, the ALDEFLUO assay illustrated that USP2 depletion significantly reduced the CSC population (Fig. 1e). Bmi1, Nanog and Oct3/4 are essential renewing and pluripotent regulators of stem cells and CSCs^18,28–30^. We next examined the gene expression of Bmi1, Nanog and Oct3/4 in relation to USP2 and found that their expression was downregulated upon USP2 deficiency (Supplementary Figure 2e). These results indicate the essential role of USP2 in the self-renewal and expansion of CSCs. Davis et al. recently reported the discovery of ML364, a specific small molecule inhibitor of USP2 identified through high throughput screening^31^. ML364 directly binds to USP2 and selectively inhibits its enzymatic activity^31^. Our finding showed the requirement of USP2’s enzymatic activity on CSC self-renewal (Supplementary Figure 1c) which then led us to test the potential effect of ML364 on CSCs. We demonstrated that pharmacological inhibition of USP2 by ML364 prominently reduced the formation of TNBC tumorspheres in a dose-dependent fashion (Fig. 1f, g). These findings collectively highlight that the inhibition of USP2 is a promising therapeutic approach to target CSCs.

### USP2 deficiency augments TNBC cells and CSCs sensitivity to chemotherapies

**Fig. 2:**
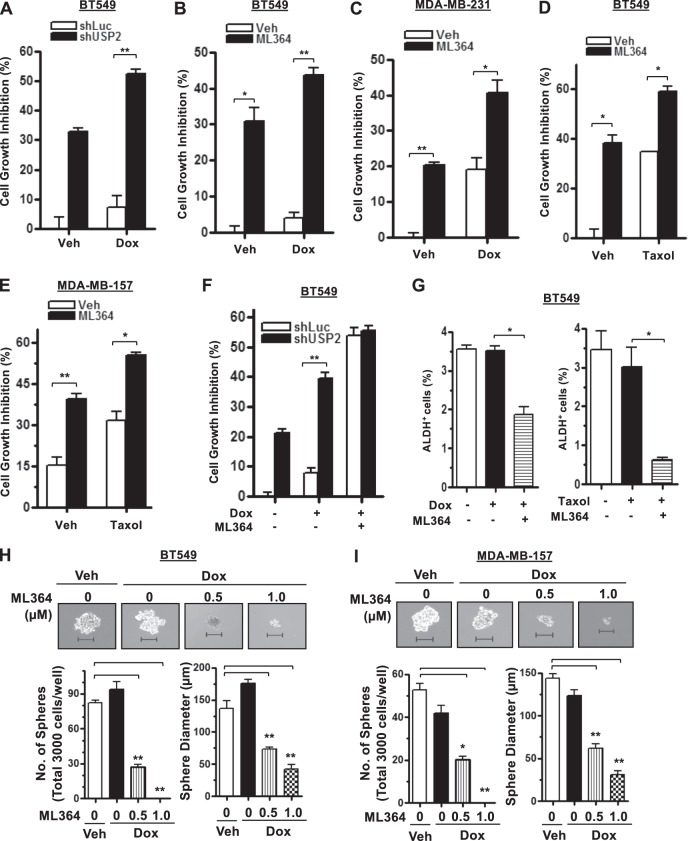
USP2 deficiency increases the sensitivity of TNBC cancer cells and CSCs to chemotherapeutic agents. **a** Cell growth inhibition assay in BT549 cells stably infected with lentivirus containing shRNA targeting Luciferase or USP2 in the absence and presence of 2 nM doxorubicin (Dox). DMSO was used as vehicle control. The number of viable cells was counted with a hemocytometer. **b**, **c** Cell growth inhibition assay in BT549 (**b**) and MDA-MB-231 (**c**) cells treated with vehicle control or the 2 μM ML364 in the absence and presence of 1 nM doxorubicin for 72 h. DMSO was used as vehicle control. The number of viable cells was counted with a hemocytometer. **d**, **e** Cell growth inhibition assay in BT549 (**d**) and MDA-MB-157 (**e**) cells treated with vehicle control or 2 μM ML364 in the absence and presence of 1 nM paclitaxel (Taxol) for 72 h. DMSO was used as vehicle control. The number of viable cells was counted by a hemocytometer. **f** Cell growth inhibition assay in Luciferase- and USP2-knockdown BT549 cells in response to the treatment of vehicle control (DMSO), doxorubicin alone, and doxorubicin in combination with ML364. **g** Quantification results for the population of ALDH^+^ cells in BT549 cells after the treatment with ML364 plus the absence and presence of doxorubicin or Taxol. **h**, **i** Representative images and quantitative results of tumorsphere formation assay treated with vehicle control or 1 nM doxorubicin in combination with various doses of ML364 in BT549 (**h**) and MDA-MB-157 (**i**) cells for 5 days. Cells were pre-treated with ML364 for 24 h before seeded. Scale bar, 100 μm. The number of spheres was counted under a light microscope; the diameter of spheres was calculated using the NIS-Element software (Nikon). Results shown in (**a**–**i**) are presented as mean value ± SEM; *p < 0.05, **p < 0.01

Doxorubicin and paclitaxel are commonly used systemic treatments for TNBC patients^32^. Even though patients diagnosed with TNBC are initially susceptible to chemotherapy, their prognosis still remains poor^33^. In light of the vital role of USP2 in CSC regulation, we sought to determine whether USP2 inhibition increases TNBC sensitivity to doxorubicin and paclitaxel. Knockdown or inhibition of USP2 alone was found to reduce TNBC cell viability (Fig. 2a–e). Moreover, USP2 inhibition by ML364 considerably enhanced the cytotoxicity of doxorubicin or paclitaxel in multiple TNBC cell models (Fig. 2a–e). To elucidate if the combinatorial effect of ML364 with doxorubicin is attributed to USP2, we treated Luciferase- and USP2-knockdown BT549 cells with ML364. We found that while ML364 treatment enhanced the efficacy of doxorubicin on Luciferase-knockdown BT549 cells, the same treatment did not further inhibit the growth of USP2-knockdown cells (Fig. 2f), underscoring that ML364 enhances doxorubicin efficacy through USP2 inhibition. The enriched CSC population in TNBC is known as a primary cause to the treatment failures due to their slow-cycling or quiescent nature. Of note, while CSCs in TNBC are intrinsically resistant to doxorubicin as anticipated, ML364 in combination with doxorubicin synergistically eliminated the CSC population in both BT549 and MDA-MB-157 cells (Fig. 2h, i). Furthermore, the ALDEFLUO assay revealed that while doxorubicin or Taxol alone did not affect the percentage of ALDH^+^ cells, ML364 in combination with either doxorubicin or Taxol effectively diminished the ALDH^+^ population (Fig. 2g). These data collectively suggest that pharmacological inhibition of USP2 can potentially be used to improve chemotherapy efficacy in TNBC.

### USP2 regulates Bmi1 by stabilizing Twist protein expression

**Fig. 3:**
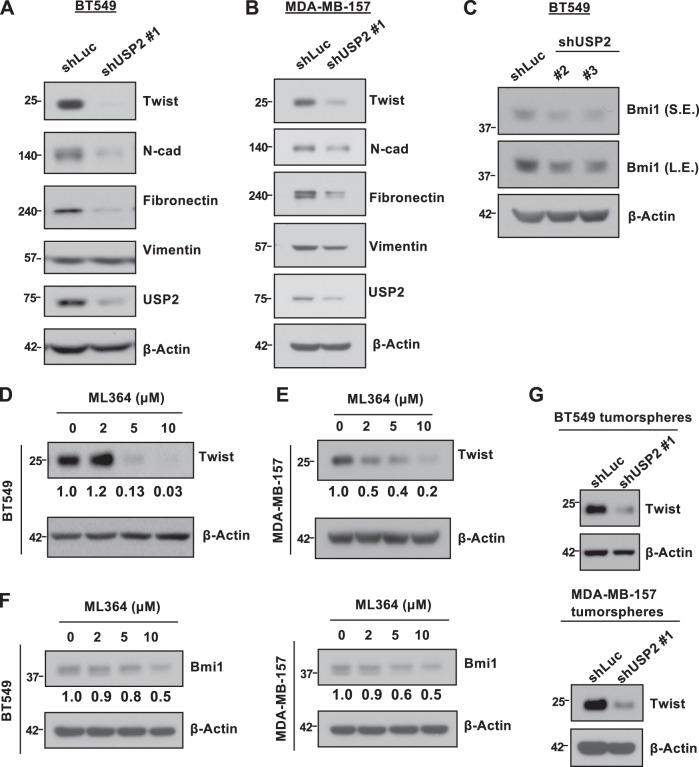
USP2 regulates the expression of Twist and Bmi1. **a**, **b** Western blot analysis for protein expression of Twist and mesenchymal markers such as N-cadherin, Fibronectin and Vimentin in BT549 (**a**) and MDA-MB-157 (**b**) cells stably infected with lentivirus containing shRNA targeting Luciferase or USP2. **c** Western blot analysis for Bmi1 protein expression in BT549 infected with lentivirus containing shRNA targeting Luciferase or USP2. Two different USP2-targeting shRNAs were used in this assay. S.E. indicates short exposure; L.E. indicates long exposure. **d**, **e** Western blot analysis for protein expression of Twist in BT549 (**d**) and MDA-MB-157 (**e**) cells treated with various doses of ML364 for 24 h. DMSO was used as vehicle control. **f** Western blot analysis for Bmi1 protein expression in BT549 and MDA-MB-157 cells treated with various doses of ML364 for 24 h. DMSO was used as vehicle control. The relative protein expression as indicated was quantified with ImageJ software and normalized to β-actin, an internal control. **g** Western blot analysis for Twist protein expression in the tumorspheres isolated from Luciferase- and USP2-knockdown BT549 and MDA-MB-157 cells

The oncogenic roles and pathological relevance of Bmi1 in human cancers have attracted considerable attention. Recent studies have demonstrated the indispensable role of Bmi1 in sustaining CSC renewal and CSC-mediated tumorigenesis in various cancer types including breast cancer^18,34^. Having shown the prominent effects of USP2 on Bmi1 gene expression and CSC self-renewal in TNBC (Supplementary Figure 2e), we subsequently aimed to decipher the molecular mechanisms underlying Bmi1 overexpression by USP2. Yang et al. and Deng et al. reported that Twist is a transcription factor of Bmi1 and that Twist overexpression increases mRNA and protein expression of Bmi1 in various cancer cells^19,20^. In line with these observations, Twist knockdown in BT549 cells reduced Bmi1 protein expression (Supplementary Figure 3a), suggesting that USP2 works through Twist for Bmi1 induction. BT549 and MDA-MB-157 TNBC cells exhibit high levels of Twist. We thus elucidated the impact of USP2 on Twist in these two cell models. Western blot assays illustrated that ablating USP2 expression in BT549 and MDA-MB-157 cells considerably reduced protein expression of Twist as expected (Fig. 3a, b and Supplementary Figure 3b). Moreover, Bmi1 protein level was found to be downregulated upon USP2 genetic silencing (Fig. 3c). In support of this notion, USP2 inhibition by ML364 led to a dose-dependent reduction in Twist and Bmi1 expression (Fig. 3d–f and Supplementary Figure 3c). It is worth noting that Bmi1 is involved in Twist-induced EMT^19^. Our western blot and real-time PCR assays demonstrated that USP2 deficiency inhibited the expression of mesenchymal markers including N-cadherin and Fibronectin in TNBC cells (Fig. 3a, b and Supplementary Figure 3d, e). Furthermore, Twist knockdown phenocopies the effect of USP2 ablation on tumorsphere formation (Supplementary Figure 3f, g). These results together highlight that USP2 is a bona fide upstream regulator of Twist and accounts for the induction of Bmi1 and EMT. To further characterize the role of USP2 in Twist protein expression in the CSC population, we analyzed Twist protein expression in the tumorspheres isolated from the Luciferase- and USP2-knockodwn BT549 and MDA-MB-157 cells. USP2 depletion was found to considerably reduce Twist protein levels in the tumorspheres (Fig. 3g). These findings collectively illustrate that USP2 regulates Twist protein expression both in the total and the CSC population of TNBC cells.

### USP2 stabilizes Twist by cleaving the proteolytic ubiquitination of Twists by β-TrCP

**Fig. 4:**
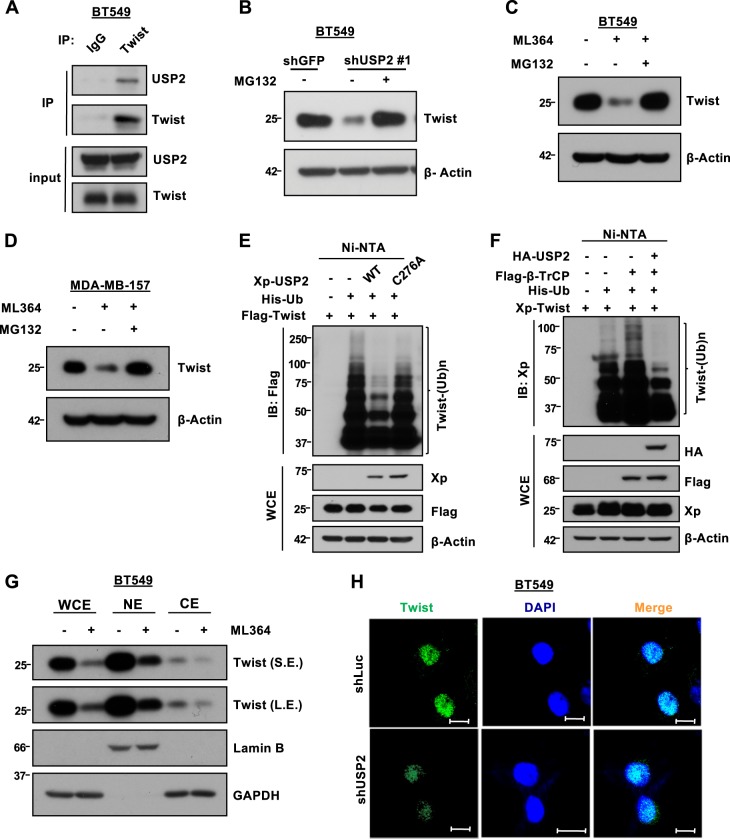
USP2 stabilizes Twist by removing ubiquitin-mediated proteasomal degradation of Twist. **a** Co-immunoprecipitation assay in BT549 cells for the interaction between endogenous USP2 and Twist protein. **b** Western blot analysis of Twist expression in BT549 cells with stable knockdown of GFP or USP2 in the absence or presence of the proteasomal inhibitor MG132 for 5 h. **c**, **d** Western blot analysis of Twist expression in BT549 (**c**) and MDA-MB-157 (**d**) cells treated with DMSO or ML364 in the absence or presence of the proteasomal inhibitor MG132 for 5 h. **e** In vivo ubiquitination assay in 293T cells transfected with Flag-tagged Twist, Histidine-tagged ubiquitin (His-Ub) and Xpress (Xp)-tagged USP2. Ni-NTA indicates nickel-nitrilotriacetic acid bead precipitate; WCE indicates whole-cell extract. MG132 was treated for 5 h before cell harvesting. **f** In vivo ubiquitination assay in 293T cells transfected with Xp-Twist, His-Ub, Flag-β-TrCP and HA-USP2. **g** Biochemical fractionation of BT549 cells in the absence or presence of the USP2 inhibitor ML364. NE stands for nuclear fraction; CE stands for cytosolic fraction. Lamin B serves as a nuclear marker and GAPDH is a cytosolic maker. The relative protein expression as indicated was quantified with ImageJ software and normalized to GAPDH or Lamin B. **h** Representative images for immunofluorescence staining of Twist subcellular localization in BT549 cells infected with lentiviruses containing shRNA targeting Luciferase or USP2. Cells were fixed, stained with indicated antibodies and 4’,6-diamidino-2-phenylindole (DAPI), and subjected to confocal microscopy analysis. Scale bar represents 10 μm. DAPI was used to stain the nucleus

We carried out a co-immunoprecipitation experiment and showed a strong interaction between endogenous USP2 and Twist in BT549 cells (Fig. 4a). Further characterization of the interaction between USP2 and Twist uncovered that the N-terminal domain of USP2 mediates its binding to Twist (Supplementary Figure 4a). To understand how USP2 affects Twist protein expression, we added proteasome inhibitor MG132 into BT549 cells to examine the involvement of proteasome-mediated degradation in this regulation. Indeed, MG132 treatment reverted Twist downregulation mediated by USP2 depletion (Fig. 4b). Likewise, reduced Twist protein expression caused by the USP2 inhibitor was rescued upon MG132 treatment in both BT549 and MDA-MB-157 cells (Fig. 4c, d). These results suggest that USP2 stabilizes Twist protein expression through preventing proteasome-mediated proteolysis. Twist is a short-lived protein whose protein stability is tightly controlled by the proteolytic polyubiquitination^35,36^. A ubiquitination assay showed that overexpression of wild-type (WT) USP2 efficiently removed Twist ubiquitination in the presence of MG132, whereas the enzymatically inactive C276A mutant of USP2 failed to trigger this deubiquitination event on Twist (Fig. 4e). β-TrCP is a primary E3 ligase that promotes Twist ubiquitination, leading to proteasomal degradation of Twist^35^. Our data showed that β-TrCP-driven Twist ubiquitination is abolished by overexpression of USP2 (Fig. 4f). On the other hand, we recently discovered that Twist is modified and regulated by K63-linked polyubiquitination^21,37^. K63-linked ubiquitination guides Twist localization to the nucleus for the activation of Twist functions in EMT and CSCs^37^. We thus examined if USP2 can potentially change subcellular localization of Twist. Biochemical fractionation assay and immunofluorescent staining demonstrated that Twist expression is downregulated upon USP2 inhibition by ML364 in both the cytosol and nucleus (Fig. 4g, h). Moreover, the ML364-mediated Twist protein reduction seen in both cytosolic and nuclear fractions was rescued upon MG132 treatment (Supplementary Figure 4b). In line with these observations, we found that ML364 treatment substantially decreased the half-life of Twist protein both in the cytosol and the nucleus (Supplementary Figure 4c). We next characterized the ubiquitin linkages of Twist removed by USP2 and found that USP2 primarily removes the K48-linked polyubiquitination of Twist (Supplementary Figure 4d). These results indicate that USP2 stabilizes Twist by antagonizing the K48-linked ubiquitination of Twist. To elucidate if USP2-regulated Twist protein expression and tumorsphere formation depends on its deubiquitinase activity, we re-expressed WT and the catalytically inactive C276A mutant of USP2 in the USP2-knockdown cells expressing the 3’-UTR-targeting shRNA, shUSP2 #1. Western blot and tumorsphere formation assays showed that the decreased Twist protein level and tumorspheres mediated by USP2 knockdown were rescued by WT but not the C276A mutant of USP2 (Supplementary Figure 4e). These findings collectively underscore that USP2 is a bona fide regulator of Twist and CSCs and that these USP2 functions depend on the deubiquitinase activity of USP2.

### USP2 regulates cancer cell migration and CSC renewals via the Twist/Bmi1 pathway

**Fig. 5:**
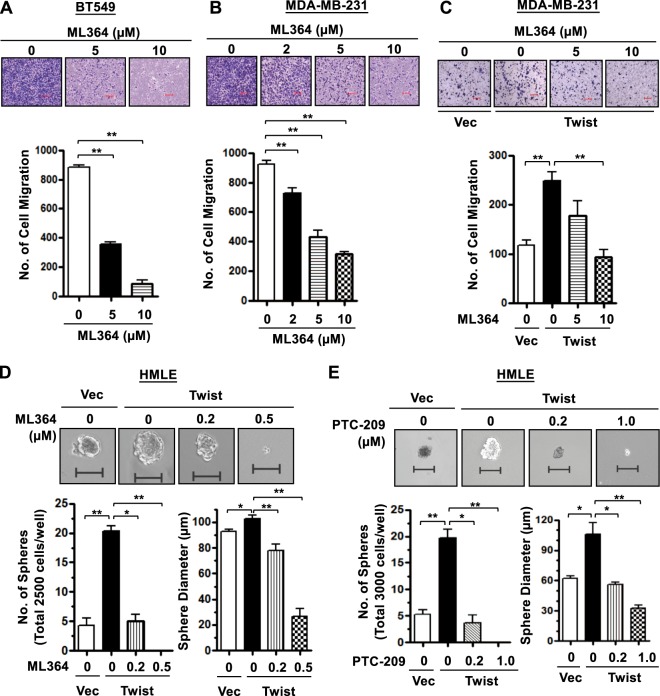
USP2 deficiency or inhibition attenuates Twist-promoted cell migration and CSC self-renewal capabilities in TNBC. **a**, **b** Representative images and quantitative results of Transwell migration assay in BT549 (**a**) and MDA-MB-231 (**b**) cells treated with various doses of ML364. Scale bar, 100 μm. **c** Representative images and quantitative results of Transwell migration assay in MDA-MB-231 cells with or without Twist overexpression followed by the treatment of various doses of ML364. Cells were allowed to migrate for 14 h. Scale bar, 100 μm. **d** Representative images and quantitative results of tumorsphere formation assay in HMLE cells with vector control or Twist overexpression followed by the treatment of various doses of ML364. Scale bar, 100 μm. **e** Representative images and quantitative results of tumorsphere formation assay in HMLE cells with vector control or Twist overexpression followed by the treatment of various doses of Bmi1 inhibitor PTC-209. Cells in (**d**, **e**) were pre-treated with ML364 or PTC-209 for 24 h before seeded. Scale bar, 100 μm. Results shown in (**a**)-(**e**) are presented as mean value ± SEM; *p < 0.05, **p < 0.01

Twist has been shown to regulate cell migration^38–40^. We next examined whether USP2 orchestrates the migration of basal-like TNBC cells. Transwell migration assays showed that knocking down USP2 expression suppressed cancer cell migration and conversely, overexpression of USP2 promoted this effect (Supplementary Figure 5a, b). In agreement with this observation, administration of ML364 resulted in a dose-dependent inhibition on the migration of BT549, MDA-MB-231, and MDA-MB-157 cells (Fig. 5a, b and Supplementary Figure 5c). Moreover, Twist-promoted cell migration and CSC renewal were downregulated in response to ML364 treatment (Fig. 5c, d and Supplementary Figure 5d), illustrating the functional link between USP2 and Twist. We next tested the effect of Bmi1 inhibition on the formation of tumorsphere induced by Twist. PTC-209 has been identified as a Bmi1-specific small molecule inhibitor that works by downregulating Bmi1 transcription^41^. We found that PTC-209 treatment effectively decreases Twist-induced tumorsphere formation (Fig. 5e), recapitulating the effect of USP2 on CSC self-renewal (Fig. 1f, g). Moreover, co-inhibition of USP2 and Bmi1 had no combinatorial effect on the number and size of tumorspheres (Supplementary Figure 5e). Our data collectively underscore that the Twist/Bmi1 pathway is a crucial mechanism that underlies USP2’s function in CSC activation.

### Pharmacological inhibition of USP2 suppresses tumor growth and augments tumor sensitivity to doxorubicin

**Fig. 6:**
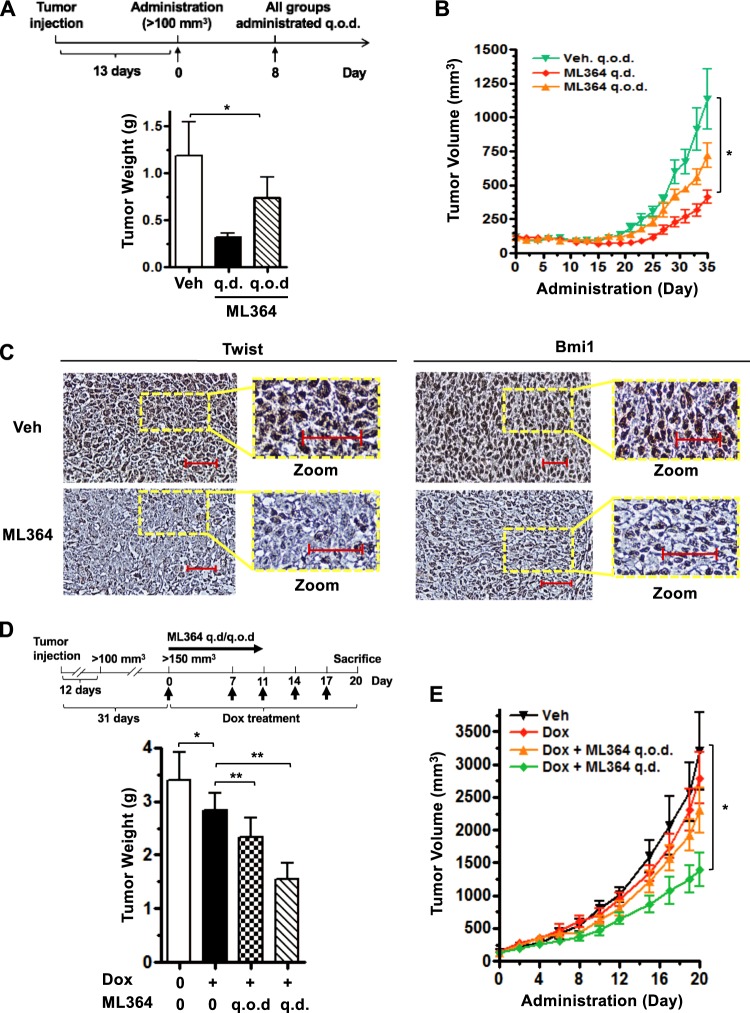
Pharmacological inhibition of USP2 suppresses tumor growth and sensitizes tumor responses to chemotherapy in pre-existing TNBC xenograft. **a**, **b** Nude mice bearing BT549 tumor xenografts were administrated with or without ML364 via i.p. injection (n = 3 per group). Treatment scheme is shown to the top of (**a**). After 35 days of administration, tumors were collected and the tumor weights are presented as mean ± SEM to the bottom of (**a**). Tumor volumes measured by caliper every other day are presented as mean ± SEM and shown in (**b**). **c** Representative images of histological analysis of Twist and Bmi1 expression in BT549-derived tumor xenografts showing that protein expression of Twist and Bmi1 is downregulated upon USP2 inhibition in vivo. Scale bar indicates 100 µm. q.o.d represents every other day and q.d. indicates each day. **d**, **e** Nude mice bearing BT549 tumor xenografts were administrated with or without ML364 in combination with doxorubicin via i.p. injection (n = 5 per group). 30 mg/kg of ML364 was administrated daily or every other day. 4 mg/kg of doxorubicin was administrated as indicated in the treatment scheme shown to the top of (**d**). After 20 days of administration, tumors were collected and the tumor weights are presented as mean ± SEM to the bottom of (**d**). Tumor volumes measured by caliper every 2–3 days as indicated are presented as mean ± SEM (**e**). Student paired t-test was used for (**a**) and (**d**); one-way ANOVA and Dunnett’s test were used to determine whether there were any statistically significant differences compared to vehicle control in (**b**, **e**). *p < 0.05, **p < 0.01

Twist has been regarded as a central driver of tumor progression and chemoresistance^42,43^. Twist depletion exhibits great potential in suppressing cancer cell proliferation and improves chemosensitivity to doxorubicin (Adriamycin)^44^. Bmi1 inhibition has been shown to enhance the sensitivity of breast cancer cells to 5-FU and radiation^45^. Since we have found that USP2 is a novel activator of the Twist/Bmi1 pathway, we subsequently determined if USP2 inhibition affects the growth and chemosensitivity of pre-existing TNBC. We first established BT549-derived xenograft in immunodeficient mice. When the tumor volume exceeds 100 mm^3^, the tumor-bearing mice were randomized for intraperitoneal injection of vehicle control or ML364. The tumorigenesis assay demonstrated that ML364 exhibited a dose-dependent suppression on both the weight and volume of BT549-derived tumors (Fig. 6a, b). We further examined the impact of ML364 on the expression of Twist and Bmi1 in vivo. Immunohistochemistry staining results illustrated that ML364 treatment in xenograft mice markedly diminished Twist and Bmi1 protein expression in tumors (Fig. 6c), supporting the conclusion that USP2 regulates the development of TNBC through the Twist/Bmi1 axis. Doxorubicin is a standard chemotherapy used to treat TNBC. Despite the potency of doxorubicin in eliminating proliferating TNBC cells, it is known to have minimal effect on the CSC population^14,46^. In light of USP2’s profound role and synergy with doxorubicin in CSC elimination (Fig. 2g, h), we strived to test the combination treatment of doxorubicin and ML364 on TNBC tumors. Our xenograft studies illustrated that while low-dose doxorubicin marginally suppressed the development of BT549-derived tumors, ML364 treatment hyper-sensitized tumor responses to doxorubicin (Fig. 6d, e).

### USP2 is upregulated in high-grade tumors and USP2 expression is correlated with lymph node metastasis in breast cancer

**Fig. 7:**
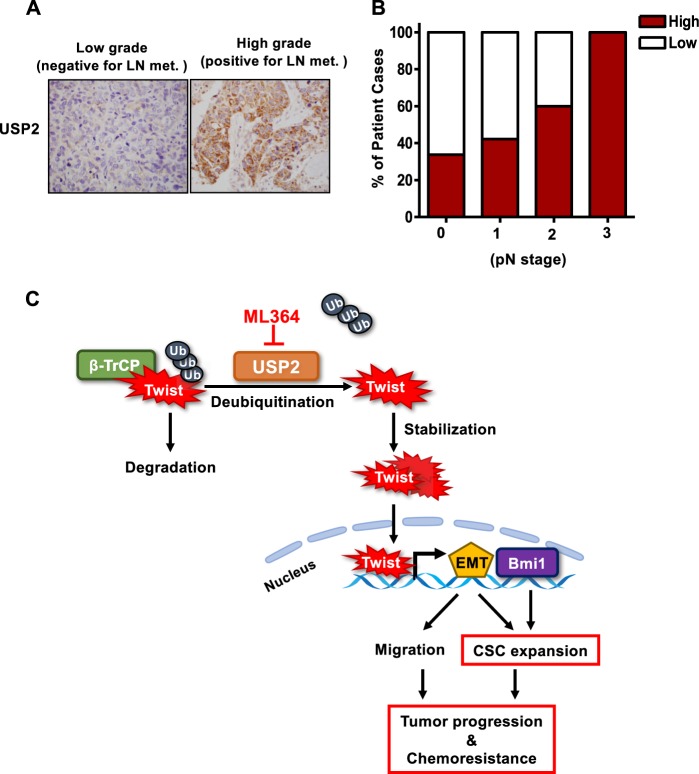
USP2 expression is correlated with lymph node metastasis in breast cancer. **a** Representative images of immunohistochemistry staining patterns for USP2 expression in resected breast tumor tissues from the patient with or without lymph node metastasis. **b** Quantitative analysis of USP2 protein expression in 223 cases of resected breast tumors in relation to different pN stages. Red bar represents the percentage of cancer cases with high USP2 expression; white bar indicates the percentage of cancer cases with low USP2 expression. **c** The working model depicts that USP2 inhibition targets CSCs and tumor growth, and improves tumor chemosensitivity by downregulating Twist/Bmi1 expression in TNBC, suggesting that USP2 is an attractive target for the development of anti-TNBC and/or CSC therapy

An advanced single-cell sequencing analysis of TNBC patient-derived xenografts revealed that CSCs accounts for the initiation of metastasis in TNBC^3^. Moreover, our data demonstrated that USP2 regulates the migratory ability of TNBC cells (Fig. 5a–c). To interrogate the potential clinical relevance of USP2 in cancer progression, we conducted immunohistochemistry staining of USP2 expression in 223 cases of breast tumor tissues. The histological analysis uncovered that USP2 expression is upregulated in breast tumors resected from patients with lymph node metastasis (Fig. 7a and Supplementary Figure 6a). Moreover, USP2 expression in breast tumors is positively correlated with the stage of lymph node metastasis (pN stage) (Fig. 7b and Supplementary Figure 6b). These results demonstrate that USP2 is correlated with breast cancer progression. Additionally, we showed that USP2 expression is positively correlated with Twist and Bmi1 expression in breast tumor specimens (Supplementary Fig. 7). The new data supports the functional connection between USP2 and Twist/Bmi1 pathway.

Altogether, our study illustrates that USP2 overexpression in TNBC promotes Twist/Bmi1 expression, CSC expansion, cancer cell migration and tumor development (Fig. 7c). We also demonstrate that inhibition of USP2 is a feasible strategy to eliminate CSCs and circumvent the chemoresistance in TNBC.

## Discussion

CSCs are primary contributors to tumor recurrence, drug resistance and progression. The CSC population is not only determined by the stem cell renewing factors but also can be replenished from differentiated cancer cells via the EMT process. Our work discovered for the first time that USP2 is a novel molecular driver of CSC self-renewal and expansion. We revealed that USP2 regulates the stem cell regulator Bmi1 and EMT, displaying pleiotropic roles in CSC modulation. USP2 regulates Bmi1 and EMT by Twist stabilization and prevents Twist ubiquitination driven by β-TrCP and subsequent proteasome-mediated protein degradation. Histological analyses uncovered the clinical relevance of USP2 in TNBC progression. Importantly, pharmacological inhibition of USP2 suppresses tumor growth and considerately improves chemosensitivity in TNBC (Fig. 7c). Our findings provide pre-clinical evidence that USP2 is an attractive target for TNBC treatment via disrupting CSC formation.

Our study supports the application of DUB inhibitors for the CSC-targeted therapy. DUBs have been demonstrated as promising targets for cancer therapy, yet their functions in CSCs are under-investigated. A few DUBs have been implicated in regulating CSC-associated transcription factors including Sox2, Bmi1, Nanog, EZH2 and ID1. Sox2 regulates the differentiation and self-renewal of CSCs. USP22 is known to bind to the promoter region of Sox2 and negatively regulates Sox2 transcription in embryonic stem cells (ESCs)^47^. The expression of USP22 is correlated with poor prognosis of several cancers. A recent report by Ma et al. demonstrated that USP22 is required for the formation of gastric stem cells by affecting the half-life of Bmi1^48^. Recent studies showed that USP21 maintains the stemness and regulates the pluripotency of ESCs by cleaving the K48-linked ubiquitin chain on Nanog, leading to Nanog stabilization^49^. USP21 is overexpressed in bladder cancer and its overexpression promotes deubiquitination and stabilization of EZH2^50^. Given the importance of Nanog and EZH2 in CSC maintenance and expansion, USP21 may potentially affect the function of CSCs. Despite the potential involvement of USP22 and USP21 in CSCs, their specific inhibitors are currently unavailable. USP1 is known to stabilize ID1 proteins, thereby preserving stem-cell traits in osteosarcoma and glioblastoma. Targeting USP1 with its selective inhibitor Pimozide displayed dramatic survival benefit in tumor-bearing mice^51^. Twist is a versatile CSC regulator, which on the one hand activates the CSC self-renewal by upregulating Bmi1 expression and on the other hand, induces the reprogramming of fully differentiated cancer cells into CSC state through EMT. Our findings that USP2 serves as a novel deubiquitinase of Twist suggest that the targeting of USP2 is a promising strategy for CSC eradication. Indeed, our results showed that genetic and pharmacological inhibition of USP2 prominently impedes Twist expression and Twist-mediated CSC characteristics including self-renewal and chemoresistance in TNBC. Importantly, administration of the USP2 inhibitor ML364 in TNBC xenograft-bearing mice restarted tumor development as a single agent and considerately improves the effectiveness of doxorubicin as a combination agent. Our work provides a rationale for further assessment of USP2 inhibitors for TNBC treatment. Of note, ML364 also inhibits USP8 to a degree similar to USP2 due to the similarity between the active sites of the two enzymes^31^. USP8 has been implicated in regulating cell migration by stabilizing connexin43, a gap junction protein^52^. Moreover, the expression levels of USP8 and Cx43 proteins are positively correlated in breast cancer specimens^52^, suggesting a potential role of USP8 in breast cancer progression. In view of Twist’s essential role in cell migration and breast cancer progression, future studies will be needed to delineate whether USP8 regulates cancer cell migration and cancer progression through Twist stabilization.

Previous studies have uncovered several oncogenic properties of USP2, including its function in regulating the protein substrates involved in survival and growth of proliferating cells. Stevenson, et al. showed that USP2 prevents cell apoptosis induced by stabilizing MDM2, in turn leading to p53 reduction^53,54^. However, our Western blot assays demonstrated that depleting USP2 expression in multiple TNBC cells neither downregulated MDM2 nor upregulated p53 expression (Supplementary Fig. 8a), suggesting that the MDM2/p53 axis is not involved in USP2’s functions in CSCs. USP2 also facilitates cell cycle transition by preventing ubiquitin-dependent degradation of cyclin D1^55^. We found that USP2 knockdown consistently downregulated cyclin D1 expression in multiple TNBC cells (Supplementary Fig. 8a). These results support our observation that USP2 knockdown suppressed the growth of TNBC cells. In agreement with these reports, we also observed growth suppression in our USP2-depleted TNBC cells (Supplementary Figure 8b, c). Aside from cyclin D1’s well-established role in cell cycle transition and cell growth, a recent study revealed that overexpression of cyclin D1 enhanced the formation of CSCs in liver cancer^56^. To date, whether and how cyclin D1 regulate CSCs in TNBC remains unclear. Since our study is the first to uncover USP2’s novel function in CSCs, future studies will be needed to delineate the contribution of cyclin D1 to USP2-mediated CSC expansion in TNBC.

To examine if USP2 has a potential to be used as a diagnostic biomarker, we analyzed USP2 expression in 223 cases of breast tumor specimens. We observed that USP2 is primarily expressed in tumor cells but not in the stroma. Moreover, upregulation of USP2 in tumor cells is significantly correlated with lymph node metastasis status and pN staging (Fig. 7a, b and Supplementary Table I). We also noticed that the percentage of breast tumor cases with high USP2 expression is increased in advanced pT status and tumor stages although the relationship is insignificant (Supplementary Table I). Since our results have shown that USP2 inhibition significantly suppresses tumor formation in TNBC, it would be interesting to examine whether the USP2 expression has any prediction power in clinicopathological parameters related to primary tumor development including pT status, tumor stages and histological grades in the TNBC subtype. Nevertheless, the information from our clinicopathological analysis suggests that USP2 could be used as a new predictive biomarker for breast cancer metastasis.

## Materials and methods

### Cell culture and reagents

BT549, MDA-MB-231 cell lines were kindly provided by Dr. MC Hung, the MDA-MB-157 cell line was kindly provided by Dr. BP Zhou, and the 293T cell line was from ATCC. Cells were cultured in DMEM containing 10% fetal bovine serum (FBS) and 1% penicillin/streptomycin and maintained in a humidified atmosphere of 5% CO_2_ at 37 °C. Cells were authenticated by short tandem repeat profiling and routinely verified to be free of mycoplasma contamination via the R&D MycoProbe® Mycoplasma Detection kit. Full length of Xpress (Xp)-tagged USP2 construct was cloned into pcDNA4 vector using pcDNA4/HisMax^©^ TOPO® TA Expression kit (Invitrogen). The enzymatically inactive C276A mutant of Xp-USP2 was generated using the QuickChange site-directed mutagenesis kit (Strategen), according to the manufacturer’s protocol. Flag-β-TrCP and Flag-Twist were gifts from Dr. MC Hung. (His)_6_-Ub and HA-USP2 constructs were from Drs. D Bohmann and W Guo, respectively. pLKO-shGFP, pLKO-shLuc and various clones of pLKO-shUSP2 constructs were from Sigma. pDEST puro HA-USP2 construct was from Addgene (#22577). The USP2 inhibitor ML364 was purchased from Axon MedChem. Doxorubicin, paclitaxel and PTC-209 were from Sigma. MG132 was from EMD Millipore.

### Western blot, viral infection, real-time PCR and nuclear fractionation

Western blot, viral infection, real-time PCR and nuclear fractionation analyses were performed as previously described^37^. Antibody, shRNA target sequence and primer sequence details are provided in Supplementary Table II-IV.

### Tumorsphere forming assay

Cells were seeded in 6-well ultra-low attachment plates (Corning) in plating medium (MEGM) in triplicate for the formation of non-adherent spheroids. The appearance of tumorspheres for HMLE, BT549, MDA-MB-157, and MDA-MB-231 cells were evaluated after 5, 6, 7 and 8 days, respectively, unless otherwise indicated. Tumorspheres with diameters ≥ 100 μm were manually counted under a microscope, unless otherwise indicated. Spherical diameters were calculated under the light microscope using NIS-Element software (Nikon). For tumorsphere isolation, cells were seeded 3 × 10^4^ cells/well in one 6-well ultra-low attachment plate in plating medium. After 10 days in culture, the tumorspheres were harvested for Western blot.

### ALDEFLUOR assay

The ALDH enzyme activity was determined by using ALDEFLUOR kit (STEMCELL Technologies) and the population of ALDH^+^ cells was quantified or sorted by flow cytometry. For quantification, cells were washed with PBS twice and suspended in ALDEFLUOR assay buffer to a concentration of 1 × 10^6^ cells/mL 0.5 mL of cell suspension were then mixed with 1 μL ALDH substrate and incubated for 40 min at 37 ℃. To serve as a gating control, 70 μL of ALDH-specific inhibitor Diethylaminobenzaldehyde (DEAB), was mixed with 0.5 mL of cell suspension prior to adding ALDH substrate. For the sorting of ALDH^+^ cells, 27 × 10^6^ cells were harvested stained as described above and sorted by flow cytometry. ALDH^+^ and ALDH^−^ cells were gated based on the DEAB negative control.

### Cell growth inhibition assay

Cell growth inhibition assay was performed as previously described^22^. BT549 and MDA-MB-231 were seeded at 5 × 10^3^ cells per well, and MDA-MB-157 cells were seeded at 8 × 10^3^ cells per well in a 12-well plate in triplicates. Twenty-four hours later, cells were treated with the specified drugs.

### Cell migration assay

The cell migration assay was performed in a 24-well transwell plate with 8-μm polyethylene terephthalate membrane filters in triplicate (Falcon cell culture insert; Becton-Dickinson) separating the lower and upper culture chambers. BT549 and MDA-MB-231 cells were seeded in the upper chamber at 8 × 10^4^ cells per well in serum-free DMEM. The bottom chamber contained DMEM with 10% FBS. Cells were allowed to migrate for 16 h, unless otherwise indicated. After the incubation period, the filter was removed, and non-migrant cells on the upper side of the filter were detached with the use of a cotton swab. Filters were fixed with 4% formaldehyde for 30 min, and cells located in the lower filter were stained with 0.1% crystal violet for 30 min and three random fields were counted. For the ML364 treatment experiments, BT549 and MDA-MB-231 cells were pre-treated with ML364 for 24 h before seeded for cell migration assays.

### Ubiquitination Assay

293T calcium phosphate transfection was performed with the desired plasmids and the cell lysates were harvested in 48 h. The lysate was then separated into two portions, one for the ubiquitination assay and one to measure protein expression level via western blot. The whole cell lysate was lysed using RIPA buffer containing protease inhibitor while the ubiquitination assay portions were lysed with Buffer A (6 M guanidine-HCl, 0.1 M Na_2_HPO_4_/NaH_2_PO_4_, 10 mM imidazole). The lysates were then sonicated at 50% amplitude for 30 s and then again for 15 s. After centrifugation at 13,000 r.p.m. for 15 min, the supernatant for both the ubiquitination portion and the whole cell lysate were transferred into new and separate eppendorf tubes. Ni-NTA beads were added into the ubiquitination portion and allowed to rotate at room temperature for 3 h while the whole cell lysate portion was mixed with 5X sample buffer and placed in −20 °C for later use. After 3 h of rotation at room temperature, beads were then washed twice with Buffer A, twice with a 1:3 ratio of Buffer A and TI Buffer (25 mM Tris-HCl, 20 mM imidazole) and once with TI Buffer. 2X sample buffer was then added to the beads and boiled for 10 min before running on an SDS-Page gel along with the whole cell lysate.

### Immunofluorescence staining

Cells were seeded on a 4-well chamber slide (Falcon) overnight, washed with PBS and fixed at −20 °C with pre-chilled methanol for 20 min. After fixation, cells were rinsed with cold acetone for three minutes and air-dried. Slides were then blocked with 10% BSA in PBS and subjected to staining with anti-Twist (Abcam; #ab50887) and DAPI. Alexa-488-conjugated goat anti-mouse secondary antibody (Molecular Probes) was applied prior to visualization of images using a Leica SP5 confocal microscope.

### Animal studies

For the xenograft tumor mouse model, 5 × 10^6^ of BT549 cells were injected into the mammary fat pad of athymic nude mice (NCr-Foxn1^nu^) purchased from Envigo. Mice were between 5 to 6 weeks old at the time of experiments. When tumor volume exceeds 100 mm^3^ or 150 mm^3^ as indicated, mice were randomly grouped for drug treatment. 30 mg/kg of ML364 was administrated every day or every other day via intraperitoneal injection. A concentration of 4 mg/kg of doxorubicin was administrated weekly via intraperitoneal injection. Tumor size was measured with a caliper, and tumor volume was calculated by using the standard formula L x W^2^ × 0.52, where *L* and *W* are the length and width, respectively. Female mice were used in this experiment. Blinding was done during tumor inoculation. All animal experiments were performed under IACUC-approved protocols (#984737). For IHC staining, tumor tissues were fixed with 10% formalin at 4 °C for one day and stored at 70% ethanol at 4 °C for two days. Tumors were then embedded in paraffin, sectioned and performed IHC staining by Stony Brook University Research Histological Core.

### Patient information and clinicopathologic parameters

Hematoxylin and eosin (H&E) stained tissue microarrays (TMAs) were purchased from US Biomax. Each single tissue spot on every array slide is individually examined by pathologists certified according to WHO published standardizations of diagnosis, classification and pathological grade. Clinicopathological data including age, tumor histologic type, lymph node status, clinical stage, pathological stage, ER, PR and Her2 status were provided by US Biomax. Each specimen collected was consented to by both hospital and individual. Discrete legal consent form was obtained and the rights to hold research uses for any purpose or further commercialized uses were waived.

### Immunohistochemistry and scoring

Commercially available unstained TMA slides were baked at 60 °C for 1 h. Following deparaffinization, rehydration, and blockage of endogenous peroxidase, slides were treated with antigen retrieval solution followed by incubation with a primary antibody against USP2 (1:50 – Proteintech). Antibody binding was visualized using the ChemMate DAKO EnVision kit (DAKO, K5001). The slides were incubated with the secondary antibody for 30 min and developed with 3,3’-diaminobenzidine for 5 min. A positive control and a negative control were carried out in each staining. TMA slides including normal breast tissue, mixed invasive breast carcinoma with different subtypes were validated to validate the proper concentration of USP2. Immunoexpression of USP2 was examined and scored by two pathologists blinded to patient clinicopathological characteristics. The staining was evaluated based on a combination of both the percentage and intensity of positively stained tumor cells to generate an H-score^57^, which was calculated using the following equation: 3 x percentage of strongly staining nuclei + 2 × percentage of moderately staining nuclei + percentage of weakly staining nuclei, giving a range of 0–300. H score less than 100 was considered low expression, and H score 100 and more were considered high expression.

### Statistical analysis

All data are shown as means ± SEM for three independent experiments, unless otherwise indicated. All statistical significance was determined by two-tailed Student’s t tests, and *p* values less than 0.05 were considered statistically significant, unless otherwise indicated. For human breast cancer samples, statistical analyses were performed using the SPSS software 22.0 (IBM Corporation, New York, NY). A Pearson’s chi-square test or Fisher’s exact test was used to compare proportions between different patient subgroups, in accord with clinicopathologic marker status. All tests were two-sided and *p* values of 0.05 or less were considered statistically significant.

## Supplementary information

Supplemental Figure 1-8

supplementary figure legends

Supplementary Table I-IV

## References

[1] Wahba HA, El-Hadaad HA (2015). Current approaches in treatment of triple-negative breast cancer. Cancer Biol. Med..

[2] Honeth G (2008). The CD44 + /CD24- phenotype is enriched in basal-like breast tumors. Breast Cancer Res..

[3] Lawson DA (2015). Single-cell analysis reveals a stem-cell program in human metastatic breast cancer cells. Nature.

[4] Batlle E, Clevers H (2017). Cancer stem cells revisited. Nat. Med..

[5] Huang X, Dixit VM (2016). Drugging the undruggables: exploring the ubiquitin system for drug development. Cell Res..

[6] Liu, P. *et al*. K63-linked polyubiquitin chains bind to DNA to facilitate DNA damage repair. *Sci. Signal*. **11**, eaar8133 (2018). 10.1126/scisignal.aar8133 PMC6434707 29871913

[7] Harrigan JA, Jacq X, Martin NM, Jackson SP (2018). Deubiquitylating enzymes and drug discovery: emerging opportunities. Nat. Rev. Drug. Discov..

[8] Fraile JM, Quesada V, Rodriguez D, Freije JM, Lopez-Otin C (2012). Deubiquitinases in cancer: new functions and therapeutic options. Oncogene.

[9] Zhao C (2016). A novel nickel complex works as a proteasomal deubiquitinase inhibitor for cancer therapy. Oncogene.

[10] Suresh B, Lee J, Kim H, Ramakrishna S (2016). Regulation of pluripotency and differentiation by deubiquitinating enzymes. Cell Death Differ..

[11] Fraile JM (2017). USP39 deubiquitinase is essential for KRAS oncogene-driven cancer. J. Biol. Chem..

[12] Peinado H, Cano A (2008). A hypoxic twist in metastasis. Nat. Cell Biol..

[13] Shi J (2014). Disrupting the interaction of BRD4 with diacetylated Twist suppresses tumorigenesis in basal-like breast cancer. Cancer Cell..

[14] Mladinich M, Ruan D, Chan CH (2016). Tackling Cancer Stem Cells via Inhibition of EMT Transcription Factors. Stem Cells Int..

[15] Mani SA (2008). The epithelial-mesenchymal transition generates cells with properties of stem cells. Cell.

[16] Jung HY, Yang J (2015). Unraveling the TWIST between EMT and cancer stemness. Cell. Stem. Cell..

[17] Lessard J, Sauvageau G (2003). Bmi-1 determines the proliferative capacity of normal and leukaemic stem cells. Nature.

[18] Chen D (2017). Targeting BMI1( + ) cancer stem cells overcomes chemoresistance and inhibits metastases in squamous cell carcinoma. Cell. Stem. Cell..

[19] Yang MH (2010). Bmi1 is essential in Twist1-induced epithelial-mesenchymal transition. Nat. Cell Biol..

[20] Deng JJ (2016). Twist mediates an aggressive phenotype in human colorectal cancer cells. Int. J. Oncol..

[21] Lee HJ, Ruan D, He J, Chan CH (2016). Two-faced activity of RNF8: what “twists” it from a genome guardian to a cancer facilitator?. Mol. Cell. Oncol..

[22] Ruan D (2017). Skp2 deficiency restricts the progression and stem cell features of castration-resistant prostate cancer by destabilizing Twist. Oncogene.

[23] Perou CM (2000). Molecular portraits of human breast tumours. Nature.

[24] Gazinska P (2013). Comparison of basal-like triple-negative breast cancer defined by morphology, immunohistochemistry and transcriptional profiles. Mod. Pathol..

[25] Kreike B (2007). Gene expression profiling and histopathological characterization of triple-negative/basal-like breast carcinomas. Breast Cancer Res..

[26] Rodriguez-Torres M, Allan AL (2016). Aldehyde dehydrogenase as a marker and functional mediator of metastasis in solid tumors. Clin. Exp. Metastas-..

[27] Weiswald LB, Bellet D, Dangles-Marie V (2015). Spherical cancer models in tumor biology. Neoplasia.

[28] Boyer LA (2005). Core transcriptional regulatory circuitry in human embryonic stem cells. Cell.

[29] Beltran AS (2011). Generation of tumor-initiating cells by exogenous delivery of OCT4 transcription factor. Breast Cancer Res..

[30] Han J (2012). RNA interference-mediated silencing of NANOG reduces cell proliferation and induces G0/G1 cell cycle arrest in breast cancer cells. Cancer Lett..

[31] Davis MI (2016). Small molecule inhibition of the ubiquitin-specific protease USP2 accelerates cyclin D1 degradation and leads to cell cycle arrest in colorectal cancer and mantle cell lymphoma models. J. Biol. Chem..

[32] O’Reilly EA (2015). The fate of chemoresistance in triple negative breast cancer (TNBC). BBA Clin..

[33] Ismail-Khan R, Bui MM (2010). A review of triple-negative breast cancer. Cancer Control.

[34] Ma J (2012). Characterization of mammary cancer stem cells in the MMTV-PyMT mouse model. Tumour Biol..

[35] Zhong J, Ogura K, Wang Z, Inuzuka H (2013). Degradation of the transcription factor Twist, an oncoprotein that promotes cancer metastasis. Discov. Med..

[36] Lander R, Nordin K, LaBonne C (2011). The F-box protein Ppa is a common regulator of core EMT factors Twist, Snail, Slug, and Sip1. J. Cell. Biol..

[37] Lee HJ (2016). The DNA damage transducer rnf8 facilitates cancer chemoresistance and progression through twist activation. Mol. Cell.

[38] Eckert MA (2011). Twist1-induced invadopodia formation promotes tumor metastasis. Cancer Cell..

[39] Yang J (2004). Twist, a master regulator of morphogenesis, plays an essential role in tumor metastasis. Cell.

[40] Yang WH (2012). RAC1 activation mediates Twist1-induced cancer cell migration. Nat. Cell Biol..

[41] Kreso A (2014). Self-renewal as a therapeutic target in human colorectal cancer. Nat. Med..

[42] Ansieau S (2008). Induction of EMT by twist proteins as a collateral effect of tumor-promoting inactivation of premature senescence. Cancer Cell..

[43] Morel AP (2012). EMT inducers catalyze malignant transformation of mammary epithelial cells and drive tumorigenesis towards claudin-low tumors in transgenic mice. PLoS Genet..

[44] Li QQ (2009). Twist1-mediated adriamycin-induced epithelial-mesenchymal transition relates to multidrug resistance and invasive potential in breast cancer cells. Clin. Cancer Res..

[45] Ren H (2016). TWIST1 and BMI1 in cancer metastasis and chemoresistance. J. Cancer.

[46] Shibue T, Weinberg RA (2017). EMT, CSCs, and drug resistance: the mechanistic link and clinical implications. Nat. Rev. Clin. Oncol..

[47] Sussman RT (2013). The epigenetic modifier ubiquitin-specific protease 22 (USP22) regulates embryonic stem cell differentiation via transcriptional repression of sex-determining region Y-box 2 (SOX2). J. Biol. Chem..

[48] Ma Y (2017). USP22 maintains gastric cancer stem cell stemness and promotes gastric cancer progression by stabilizing BMI1 protein. Oncotarget.

[49] Jin J (2016). The deubiquitinase USP21 maintains the stemness of mouse embryonic stem cells via stabilization of Nanog. Nat. Commun..

[50] Chen Y, Zhou B, Chen D (2017). USP21 promotes cell proliferation and metastasis through suppressing EZH2 ubiquitination in bladder carcinoma. Onco. Targets Ther..

[51] Lee JK (2016). USP1 targeting impedes GBM growth by inhibiting stem cell maintenance and radioresistance. Neuro. Oncol..

[52] Sun J (2018). The ubiquitin-specific protease USP8 deubiquitinates and stabilizes Cx43. J. Biol. Chem..

[53] D’Arcy P, Wang X, Linder S (2015). Deubiquitinase inhibition as a cancer therapeutic strategy. Pharmacol. Ther..

[54] Stevenson LF (2007). The deubiquitinating enzyme USP2a regulates the p53 pathway by targeting Mdm2. EMBO J..

[55] Shan J, Zhao W, Gu W (2009). Suppression of cancer cell growth by promoting cyclin D1 degradation. Mol. Cell.

[56] Xia W (2017). Smad inhibitor induces CSC differentiation for effective chemosensitization in cyclin D1- and TGF-beta/Smad-regulated liver cancer stem cell-like cells. Oncotarget.

[57] Ishibashi H (2003). Sex steroid hormone receptors in human thymoma. J. Clin. Endocrinol. Metab..

