# Supplementary material for: Inhibition of USP2 eliminates cancer stem cells and enhances TNBC responsiveness to chemotherapy
Source: Cell Death Dis. 2019 Mar 28;10(4):285. doi: 10.1038/s41419-019-1512-6 (PMC6437220; doi:10.1038/s41419-019-1512-6)
Supplement: Supplementary file 1 — Supplemental Figure 1-8 [file 41419_2019_1512_MOESM1_ESM.pdf]

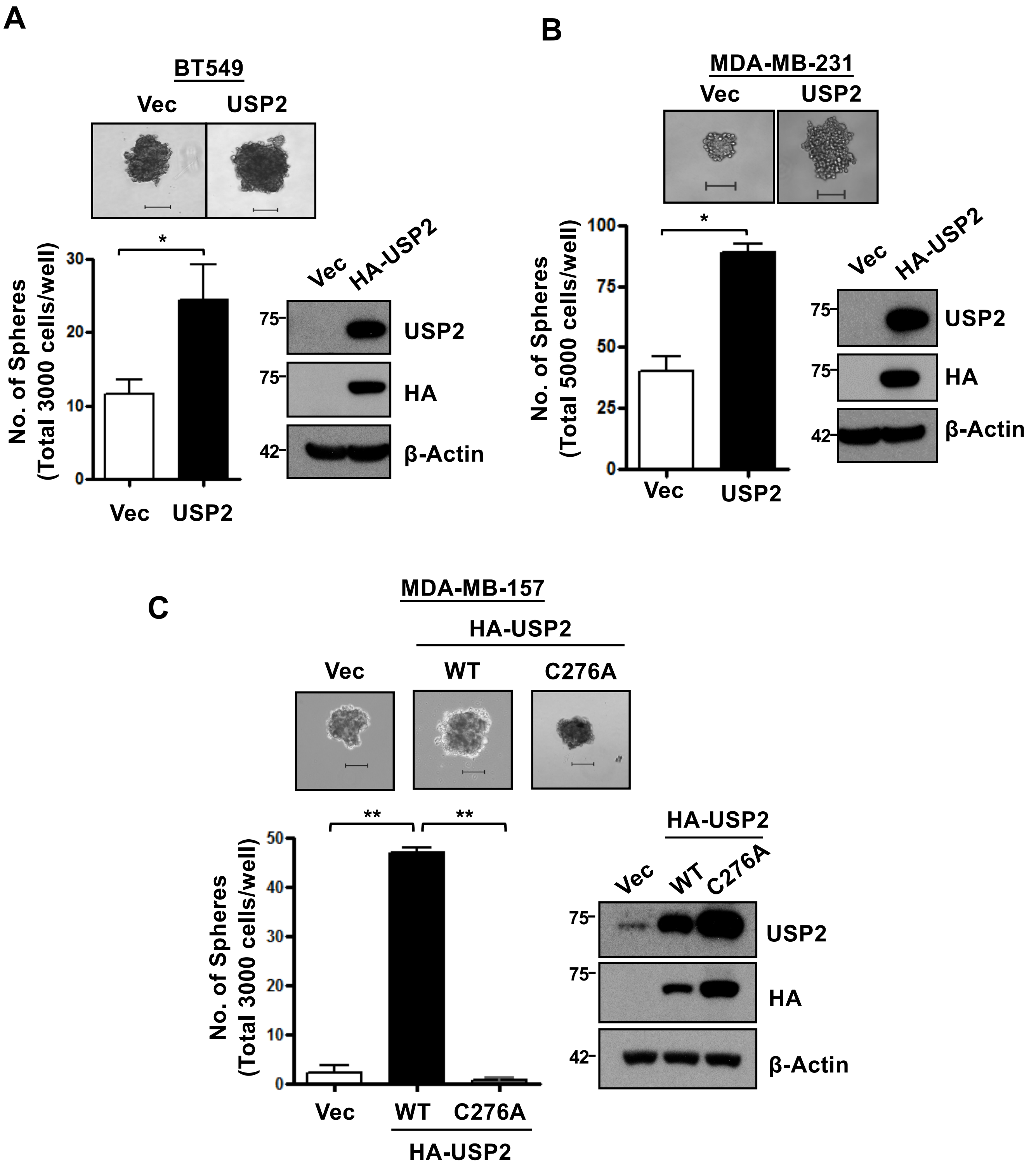

Supplemental Figure 1

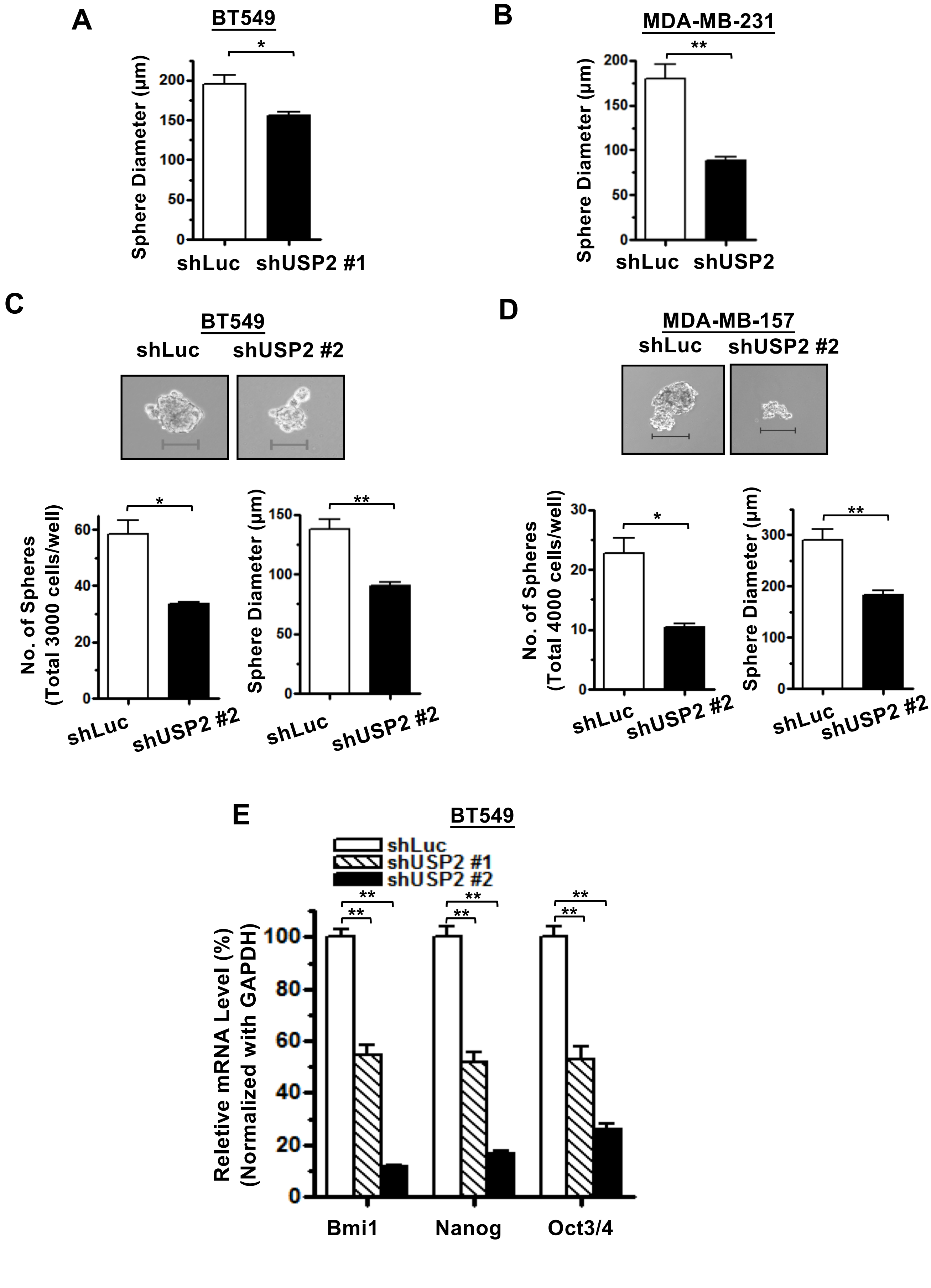

Supplemental Figure 2

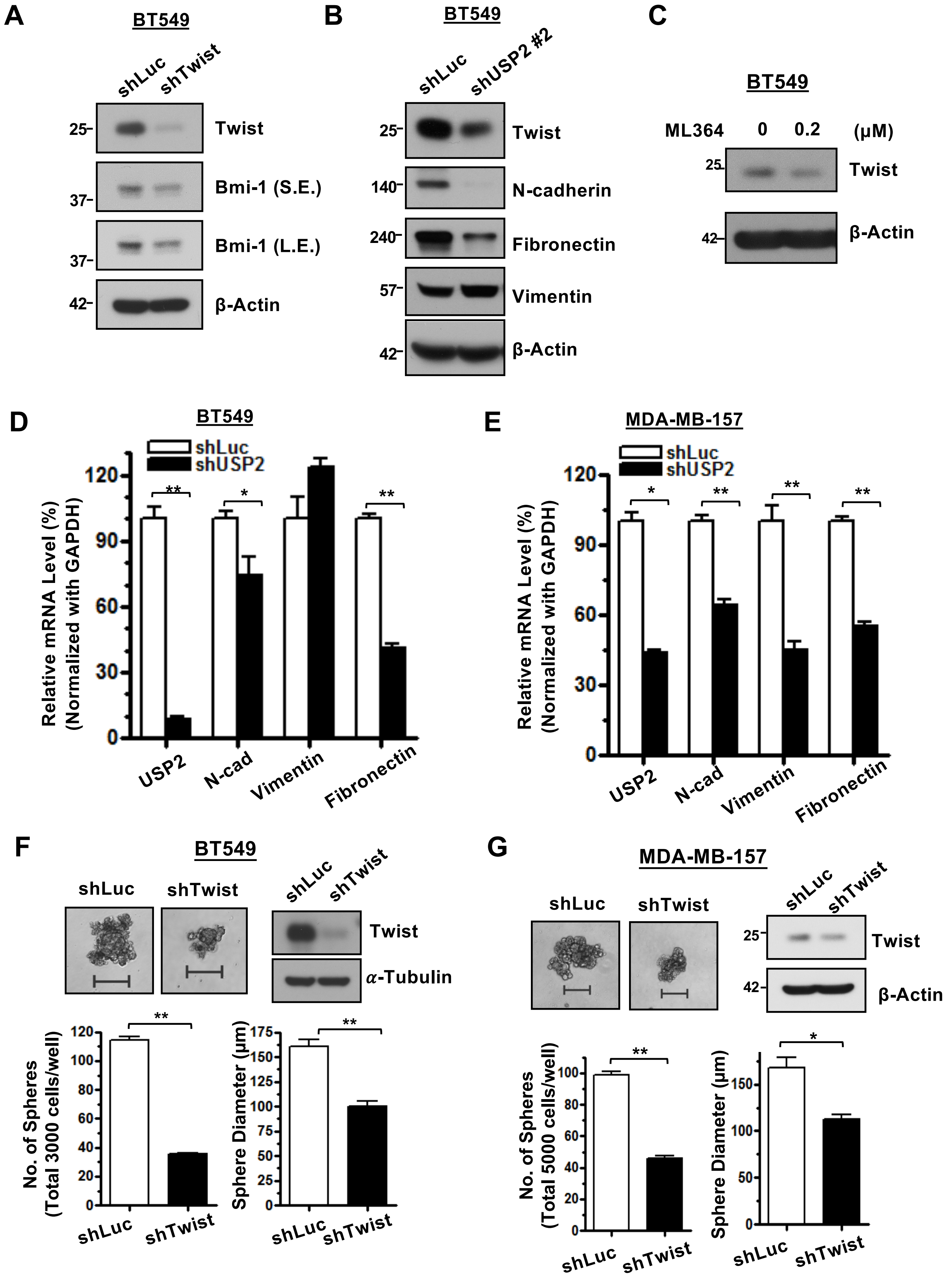

Supplemental Figure 3

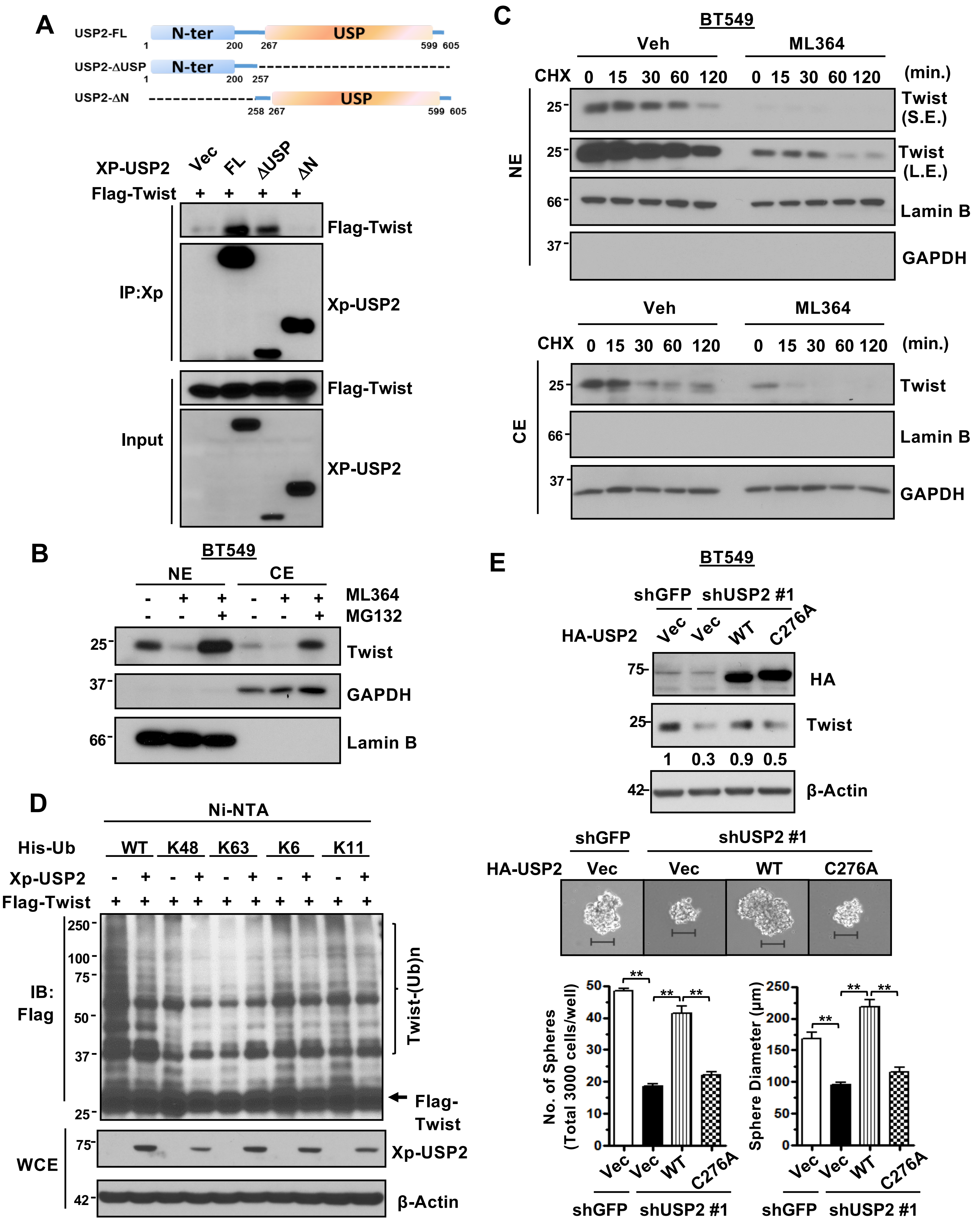

Supplemental Figure 4

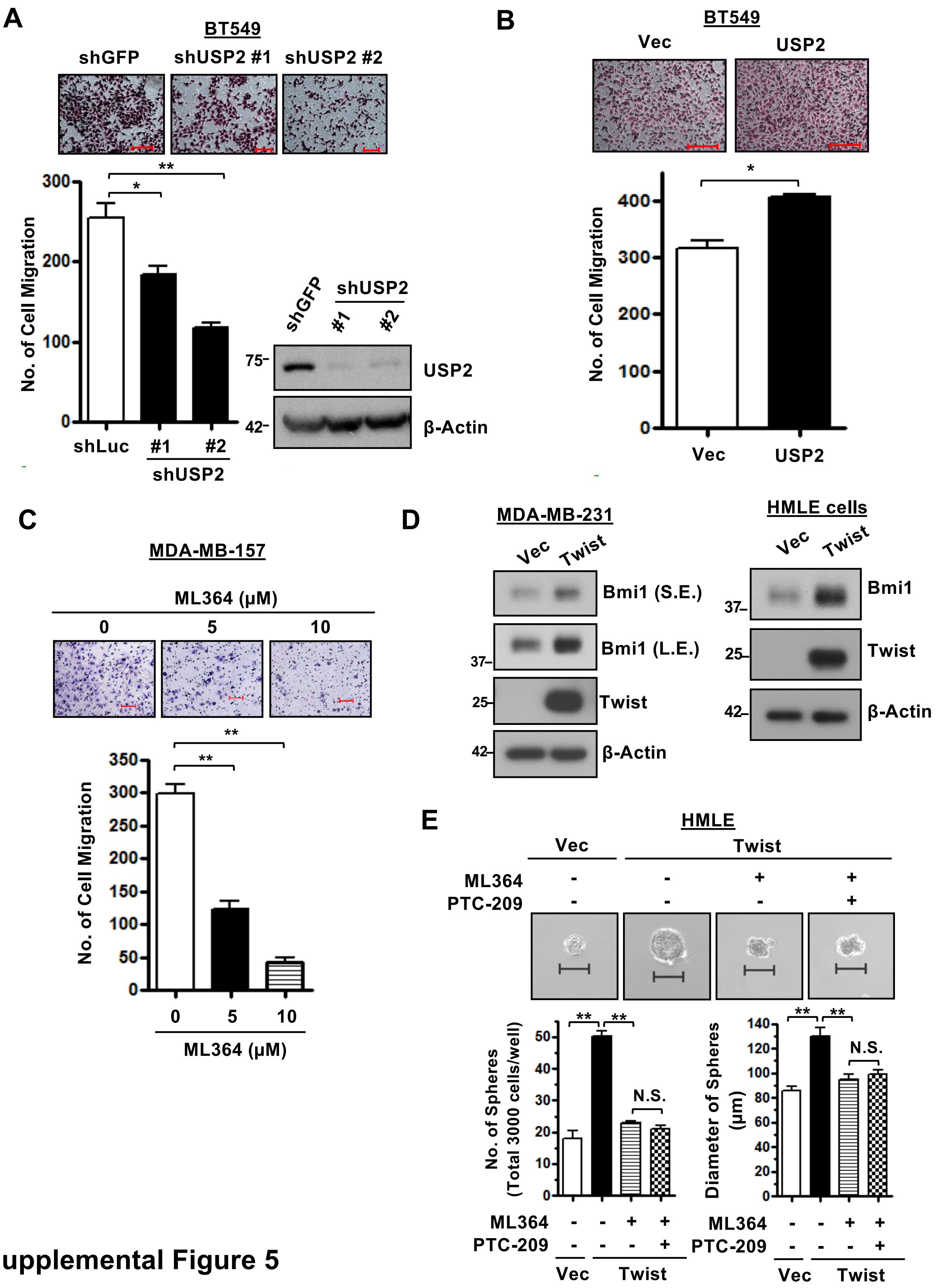

Supplemental Figure 5

A

Count		USP2_100		Total
		<100	>=100	
N_01	Negative	106	54	160
	Positive	32	31	63
Total		138	85	223

Chi-Square Tests

	Value	df	Asymptotic Significance (2-sided)	Exact Sig. (2-sided)	Exact Sig. (1-sided)
Pearson Chi-Square	4.578[a]	1	**0.032**		
Continuity Correction[b]	3.946	1	0.047		
Likelihood Ratio	4.509	1	0.034		
Fisher's Exact Test				**0.046**	**0.024**
Linear-by-Linear Association	4.558	1	0.033		
N of Valid Cases	223				

a. 0 cells (0.0%) have expected count less than 5. The minimum expected count is 24.01.

b. Computed only for a 2x2 table

B

**Count**		USP2_100		Total
		<100	>=100	
pN	N0	106	54	160
	N1	26	19	45
	N2	6	9	15
	N3	0	3	3
Total		138	85	223

Chi-Square Tests

	Value	df	Asymptotic Significance (2-sided)
Pearson Chi-Square	9.531[a]	3	**0.023**
Likelihood Ratio	10.349	3	0.016
N of Valid Cases	223		

a. 2 cells (25.0%) have expected count less than 5.
The minimum expected count is 1.14.

Supplemental Figure 6

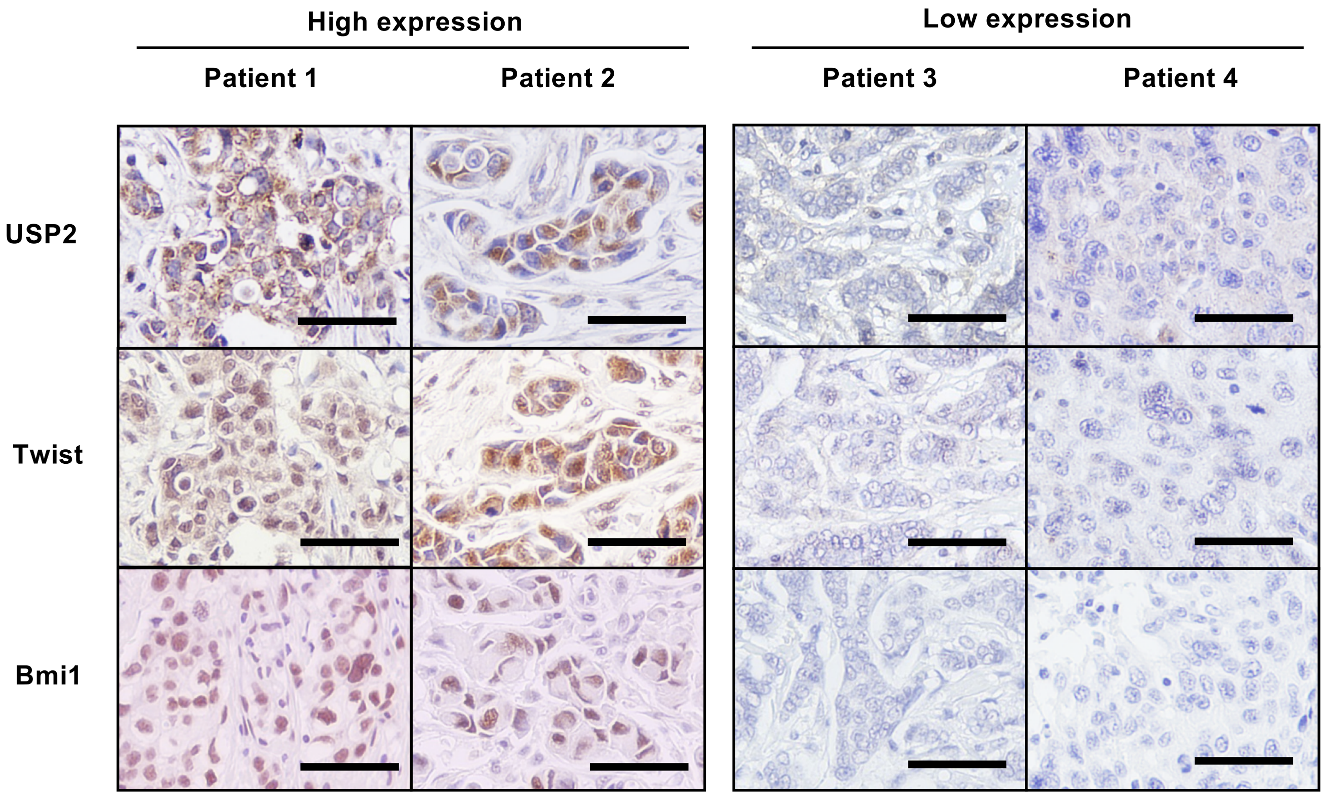

Supplemental Figure 7

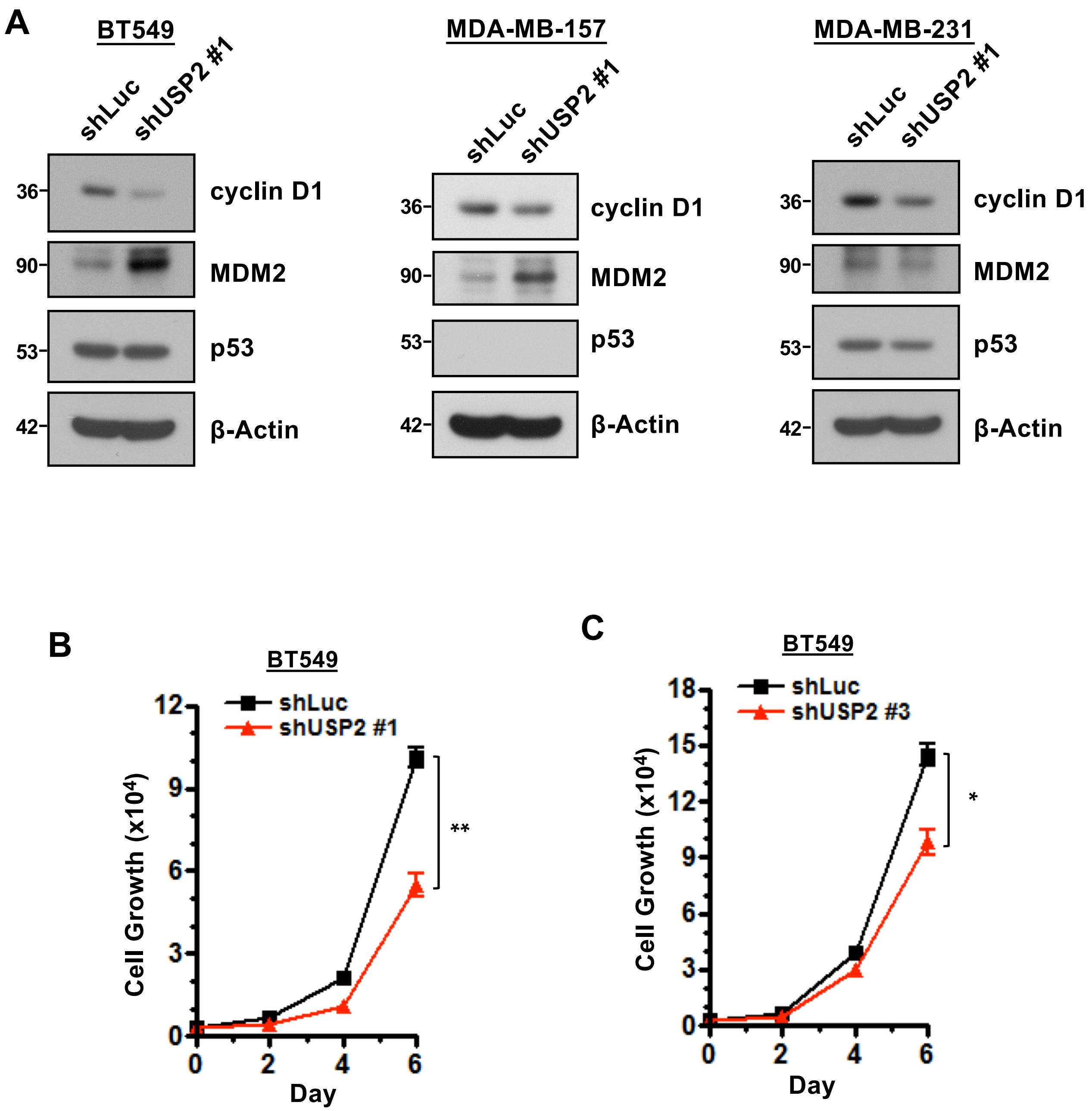

Supplemental Figure 8
