# Supplementary material for: Inhibition of USP2 eliminates cancer stem cells and enhances TNBC responsiveness to chemotherapy
Source: Cell Death Dis. 2019 Mar 28;10(4):285. doi: 10.1038/s41419-019-1512-6 (PMC6437220; doi:10.1038/s41419-019-1512-6)
Supplement: Supplementary file 2 — supplementary figure legends [file 41419_2019_1512_MOESM2_ESM.doc]

**Supplementary Information**

**Supplementary Figure 1. The enzymatic activity of USP2 is required for USP2-promoted self-renewal property of CSCs in TNBC.** (**a, b**) Representative images and quantitative results of tumorsphere formation assay in BT549 (**a**) and MDA-MB-231 (**b**) cells with overexpression of vector control or USP2. (**c**) Representative images and quantitative results of tumorsphere formation assay in MDA-MB-157 cells with overexpression of vector control, wild-type USP2 or the enzymatically inactive (C276A) mutant of USP2. Scale bar, 100 μm. Results are presented as mean value ± SEM; ** p <0.05, ** p< 0.01*.

**Supplementary Figure 2. Deficiency of USP2 suppresses the formation and tumorspheres and expression of self-renewal regulators in TNBC.** (**a, b**) Diameter analysis for the size of tumorspheres in BT549 (**a**) and MDA-MB-231 (**b**) cells stably infected with lentivirus containing shRNA targeting Luciferase or USP2. (**c**) Representative images and quantitative results of tumorsphere formation assay in BT549 cells stably infected with lentivirus containing shRNA targeting Luciferase or USP2. The USP2-targeting shRNAs used in this experiment and Figure 1c target different regions of USP2. Scale bar, 100 μm. (**d**) Representative images and quantitative results of tumorsphere formation assay in MDA-MB-157 cells stably infected with lentivirus containing shRNA targeting Luciferase or USP2. Scale bar, 200 μm. (**e**) Real-time PCR analysis of mRNA levels of Bmi1, Nanog and Oct3/4 in Luciferase and USP2-knockdown BT549 cells. Two different USP2-targeting shRNAs were used in this experiment. Results shown are presented as mean value ± SEM; ** p <0.05, ** p< 0.01*.

**Supplementary Figure 3. USP2 regulates the expression of Twist, Bmi1 and mesenchymal markers in TNBC cells.** (**a**) Western blot analysis for Bmi1 protein expression in BT549 cells stably infected with lentivirus containing shRNA targeting Luciferase or Twist. (**b**) Western blot analysis for protein expression of Twist and mesenchymal markers such as N-cadherin, Fibronectin and Vimentin in BT549 cells stably infected with lentivirus containing shRNA targeting Luciferase or USP2. The USP2-targeting shRNAs used here and Figure 3a target different regions of USP2. (**c**) Western blot analysis for Bmi1 protein expression in BT549 cells treated with vehicle control or ML364 for 7 days. (**d, e**) Real-time PCR analysis of mRNA expression for USP2 and mesenchymal marker such as Fibronectin, N-cadherin and Vimentin in BT549 (**d**) and MDA-MB-157 (**e**) cells stably infected with lentivirus containing shRNA targeting Luciferase or USP2. (**f, g**) Tumorsphere formation and Western blot assays in BT549 (**f**) and MDA-MB-157 (**g**) cells stably infected with lentivirus containing shRNA targeting Luciferase or Twist. Scale bar, 100 μm. Results shown are presented as mean value ± SEM; ** p <0.05, ** p< 0.01*.

**Supplementary Figure 4. USP2 antagonizes K48-linked ubiquitination and protein degradation of Twist.** (**a**) co-immunoprecipitation assay in 293T cells transfected with Flag-Twist and various Xp-USP2 constructs. (**b**) Biochemical fractionation of BT549 cells after the treatment of ML364 with or without MG132. NE stands for nuclear fraction; CE stands for cytosolic fraction. Lamin B serves as a nuclear marker and GAPDH is a cytosolic maker. (**c**) Biochemical fractionation of vehicle- and ML364-treated BT549 cells after incubation with cycloheximide (CHX) for various time periods. S.E. = short exposure, L.E. = long exposure. (**d**) *In vivo* ubiquitination assay in 293T cells transfected with Flag-Twist, Xp-USP2 and various linkage-specific ubiquitin constructs. Cells were treated with MG132 for 5 hours before harvesting. (e) Western blot and tumorsphere formation assays in GFP- and USP2-kncockdown BT549 cells (by the 3’-UTR-targeting shRNA, shUSP2 #1) with re-expression of vector control, WT, or the C276A mutant of USP2. The relative protein expression as indicated was quantified with ImageJ software and normalized to β-actin, an internal control.

**Supplementary Figure 5. USP2 regulates the migratory ability of TNBC cells.** (**a**) Representative images and quantitative results of Transwell migration assay in BT549 cell infected with viruses containing shRNA targeting GFP or USP2. Two different USP2-targeting shRNAs were used in this assay. Western blot results verified the knockdown efficiency of USP2 in BT549 cells. (**b**) Representative images and quantitative results of Transwell migration assay in BT549 with vector control or USP2 overexpression. (**c**) Representative images and quantitative results of Transwell migration assay in MDA-MB-157 treated with various doses of ML364. (**d**) Western blot analysis for Bmi1 protein expression in MDA-MB-231 and HMLE with vector control or Twist overexpression. (**e**) Representative images and quantitative results of tumorsphere formation assay in HMLE cells with vector control or Twist overexpression followed by the treatment of ML364 with or without PTC-209. Scale bar, 100 μm. Results shown are presented as mean value ± SEM; ** p <0.05, ** p< 0.01*, N.S., not significant.

**Supplementary Figure 6. USP2 expression is positively correlated with lymph node metastasis.** (**a**) Clinicopathological analysis of USP2 expression in relation to lymph node metastasis in 223 cases of resected TNBC tumors. Pearson’s Chi-Square test and Fisher’s Exact test were used to assess the significance of the correlation. (**b**) Clinicopathological analysis of USP2 expression in relation to different pN stages in 223 cases of resected TNBC tumors. Pearson’s Chi-Square test was used to assess the significance for the correlation.

**Supplementary Figure 7. USP2 expression is positively correlated with Twist and Bmi1 protein expression in breast tumor specimens.** Immunohistochemistry staining for the expression levels of Twist and Bmi1 in breast cancer patients with high or low USP2 expression. Scale bar, 50 μm.

**Supplementary Figure 8. USP2 depletion suppresses cancer cell growth in TNBC cells.** (**a**) Western blot analyses for protein expression of cyclin D1, MDM2 and p53 in various TNBC cells with Luciferase or USP2 knockdown. (**b**) Cell growth assay showed that USP2 knockdown inhibits the growth of BT549 cells. 2500 of Luciferase or USP2-knockdown BT549 cells were seeded and cultured in complete DMEM for 6 days. (**c**) Cell growth assay showed that USP2 knockdown inhibits the growth of BT549 cells. 3000 of Luciferase or USP2-knockdown BT549 cells were seeded and cultured in complete DMEM for 6 days. The shRNAs used in (**b**) and (**c**) target different regions of USP2.
