# Supplementary material for: Inhibition of USP2 eliminates cancer stem cells and enhances TNBC responsiveness to chemotherapy
Source: Cell Death Dis. 2019 Mar 28;10(4):285. doi: 10.1038/s41419-019-1512-6 (PMC6437220; doi:10.1038/s41419-019-1512-6)
Supplement: Supplementary file 3 — Supplementary Table I-IV [file 41419_2019_1512_MOESM3_ESM.docx]

**Supplementary Table I. Association of USP2 expression with clinicopathological features of breast tumor cases.**

|  |  | **All cases** | **Low USP2 expression <100** | **High USP2 expression**  **≥ 100 (%)** | **Significance by Pearson Chi-Square Test** | **Significance by**  **Fisher's Exact Test (2-sided/1-sided)** |
| --- | --- | --- | --- | --- | --- | --- |
| **Age (yrs)** | < 50 | 223 | 70 | 42 | 0.849 |  |
|  | ≥ 50 |  | 68 | 43 |  |  |
|  |  |  |  |  |  |  |
| **LNM^#^** | Negative | 160 | 106 | 54 (34%) | **0.032** | **0.046/0.024** |
|  | Positive | 63 | 32 | 31 (49%) |  |  |
|  |  |  |  |  |  |  |
| **pN staging** | N0 | 160 | 106 | 54 (34%) | **0.023** |  |
|  | N1 | 45 | 26 | 19 (42%) |  |  |
|  | N2 | 15 | 6 | 9 (60%) |  |  |
|  | N3 | 3 | 0 | 3 (100%) |  |  |
|  |  |  |  |  |  |  |
| **Stage** | I | 6 | 5 | 1 (17%) | 0.486 |  |
|  | II | 159 | 99 | 60 (38%) |  |  |
|  | III | 58 | 34 | 24 (41%) |  |  |
|  |  |  |  |  |  |  |
| **pT status** | Early  (T1+T2) | 143 | 90 | 53 (37%) | 0.665 | 0.669/0.385 |
|  | Late  (T3+T4) | 80 | 48 | 32 (40%) |  |  |

^#^ indicates lymph node metastasis

| **Supplementary Table II.** Target sequences used in this study | |
| --- | --- |
| *Target* | *shRNA sequence* |
| USP2 (#1) | 5'-CCTCGGCGTTTGCATTTGTAA-3' |
| USP2 (#2) | 5'-CCGCGCTTTGTTGGCTATAAT-3' |
| USP2 (#3) | 5'-CCATGCTGTTTACAACCTGTA-3' |
| Twist | 5’-CCTGAGCAACAGCGAGGAAGA-3’ |
| GFP | 5'-GCAAGCTGACCCTGAAGTTC-3' |
| Abbreviation: USP2, ubiquitin carboxyl-terminal hydrolase 2; GFP, green fluorescent protein; shRNA, short hairpin RNA. | |

| **Supplementary Table III.** Antibodies used in this study | | |
| --- | --- | --- |
| *Primary Antibody* | *Source* | *Dilution* |
| USP2 – WB | Cell Signaling  Bio-Rad | 1:2000  1:2000 |
| USP2 – IHC | Proteintech | 1:50 |
| Flag | Sigma | 1:2000 |
| Xpress | Invitrogen | 1:5000 |
| HA | Convance | 1:5000 |
| GRP78 | BD Biosciences | 1:10,000 |
| β-Actin | Sigma | 1:20,000 |
| GAPDH | Santa Cruz | 1:5000 |
| N-cadherin | Cell Signaling | 1:1000 |
| E-cadherin | Cell Signaling | 1:3000 |
| Vimentin | Cell Signaling | 1:2000 |
| Twist – WB | Abcam | 1:1000 |
| Twist – IHC | Biorbyt | 1:1500 (mouse tumors)  1:50 (human tumor tissues) |
| Fibronectin | BD Biosciences | 1:1000 |
| Bmi1-WB | Cell Signaling | 1:2000 |
| Bmi1-IHC | Cell Signaling | 1:500 (mouse tumors)  1:50 (human tumor tissues) |
| Lamin B | Santa Cruz | 1:5000 |

| **Supplementary Table IV.** Primer sequences used in this study | |
| --- | --- |
| *Target (F/R)* | *Primer sequence (5’ to 3’)* |
| USP2-F | 5’-CGGCCAGCGAGTCTACTTG-3’ |
| USP2-R | 5’-AAGGTCAAATCCGCCTTCAAC-3’ |
| GAPDH-F | 5’-GATTCCACCCATGGCAAATTC-3’ |
| GAPDH-R | 5’-CTTCTCCATGGTGGTGAAGAC-3 |
| Fibronectin-F | 5’-AAACTTGCATCTGGAGGCAAACCC-3’ |
| Fibronectin-R | 5’-AGCTCTGATCAGCATGGACCACTT-3’ |
| N-cad-F | 5’- AGCCTGACACTGTGGAGCCT-3’ |
| N-cad-R | 5’- GGAGTCATATGGTGGAGCTGT-3’ |
| Vimentin-F | 5’-AAAGTGTGGCTGCCAAGAACCTGC-3’ |
| Vimentin-R | 5’-ACTCAGTGGACTCCTGCTTTGCCT-3’ |
| Bmi1-F | 5’-CCTGGAGAAGGAATGGTCCACTTCC-3’ |
| Bmi1-R | 5’-GGTGGAGGGAATACCTCCTGCTGG-3’ |
| Nanog-F | 5’-CAACTGGCCGAAGAATAGCAATGGT-3’ |
| Nanog-R | 5’-GTCTGAGTGTTCCAGGAGTGGTTGC-3’ |
| Oct3/4-F | 5’-CTTGAATCCCGAATGGAAAGGG-3’ |
| Oct3/4-R | 5’-CCTTCCCAAATAGAACCCCCA-3’ |
